# CPA governance restructuring and section autonomy: a case study in organizational resistance to structural diversity

**DOI:** 10.1186/s13033-026-00722-z

**Published:** 2026-07-30

**Authors:** Sonya C. Faber, Amy Bartlett, Monnica T. Williams, Jude M. Cénat

**Affiliations:** 1 School of Psychology, University of Ottawa, 136 Jean-Jacques Lussier, Vanier Hall, Ottawa, ON K1N 6N5 Canada; 2 Department of Classics and Religious Studies, University of Ottawa, Ottawa, Canada

**Keywords:** Power hoarding, Organizational change, Governance, Power dynamics, Equity, Diversity, Inclusion, Professional ethics, Organizational psychology

## Abstract

This case study critically examines governance changes enacted within the Canadian Psychological Association (CPA) following internal advocacy for structural representation of Black, Indigenous, and People of Color (BIPOC) members. Drawing on qualitative document analysis, the study examines a series of bylaw amendments proposed and approved by the organization’s leadership that centralize authority within the Board and Chief Executive Officer while reducing the autonomy of constituent member groups (CPA Sections). Using the lenses of power hoarding and the division of powers framework, the analysis explores how governance reforms may reshape organizational authority, representation, and accountability. The findings illustrate how the amendments systematically shifted decision-making power away from Sections and toward central leadership, despite emerging in a context of explicit calls for greater structural diversity and power sharing. As such, the reforms can reasonably be understood as a form of organizational resistance to structural diversity. This case contributes to understanding how governance reforms may influence equity initiatives within professional organizations and offers practical implications for organizational governance, diversity consulting, and institutional accountability.

**Supplementary Information:**

The online version contains supplementary material available at 10.1186/s13033-026-00722-z.

## Introduction

### Case description: the proposed governance amendments

The Canadian Psychological Association (CPA) was founded in 1939, and according to its website, is ‘a society that values and applies psychological science for the benefit of persons, communities, organizations, and peoples’ (https://cpa.ca/who-we-are)*.* To enact this vision, the CPA has committed to serving ‘the public and the CPA’s membership by advancing psychological science, practice, and education through research, advocacy, and collaboration’. Building upon the foundations of their 2020–2025 active cycle, the CPA’s current 2025–2030 Strategic Plan continues to refine this approach by envisioning ‘a society where understanding of diverse human needs, behaviours and aspirations drive legislation, policies and programs for individuals, organizations and communities’ (CPA [11]). And that focus on diversity and inclusion is reflected in the strategic priorities of the subsequent 2025–2030 Strategic Plan, which explicitly commits the association to utilizing ‘calls to action and justice’ to support Indigenous knowledge and maintain accountability to reconciliation (CPA [12]). This includes a commitment to “Support, promote, and grow a profession, discipline, and association that is diverse, equitable, inclusive, and accessible.”

Despite these renewed strategic commitments and vision for the organization and psychological sector in Canada, in 2025 the leadership of the organization proposed a new set of bylaw amendments, stating that the changes were intended to improve administrative efficiency and align CPA governance with recognized best practices (Supplementary Materials 1 and 2). These proposals arrived shortly after a public commentary from several Section leaders, including those representing BIPOC membership, calling for designated seats on the central Board to improve structural diversity (Faber, Cenat et al. [21]).

### About the organizational structure

According to the CPA website, the vision, policies and priorities of the organization are upheld according to the current Strategic Plan. The Board of Directors lead the CPA’s three pillar committees (Professional Affairs, Scientific Affairs, Education and Training). In addition to members at large and executive positions on the Board, four non-voting seats are held by representatives from the CPA’s partner organizations, and importantly for the purposes of this commentary, two of the core Board seats were reserved for a representative of the CPA’s Student Section and another for a representative of the Council of the CPA’s Sections.

CPA Sections hold official status under the Association’s By-Laws and serve as the primary mechanism for addressing the specialized needs and interests of members. Each Section has the authority to initiate and undertake activities relevant to its members, develop position papers and policy statements within its area of expertise, and organize meetings as part of CPA’s annual convention. Sections may also make specific representations to external agencies or organizations with the Board of Directors’ approval or recommend that CPA itself undertake such representations. Through these powers, the Sections play a vital role in the governance of the organization, helping to ensure CPA is meeting its strategic priorities, as well as advancing the profession and ensuring that diverse areas of psychological practice and research are supported within the Association.

Despite this structure and strategic priorities, there have been concerns that the organization is not meeting its stated goals, and there have been calls for more equity in governance for some time, marked by recent fears over an EDI rollback [24, 36]. This is not a new issue facing the CPA. Over six years ago in 2019, a critical call for greater representation was made regarding Responding to the Needs of First Nations and Underrepresented Groups, emerging at the National Summit on the Future of Professional Psychology Training [38]. It was agreed that, “At all decision-making tables, including boards, task forces and committees, there must be substantive representation of community-based persons of both Indigenous and broader Equity, Diversity, and Inclusion perspectives,” with an overwhelming majority in favor, rated at a high level of urgency (p. 239). This recommendation did not escape the notice of the CPA, given that the lead author of the paper reporting on this decision was the CPA president at the time. However, this directive was never implemented, and renewed calls for diversification of the Board marked a tipping point.

The by-law amendments do the opposite of what was being requested, as they centralize additional authority within the Board. The composition of that governing body provides important context for understanding concerns regarding representation and participation in organizational decision making.

**Fig. 1:**
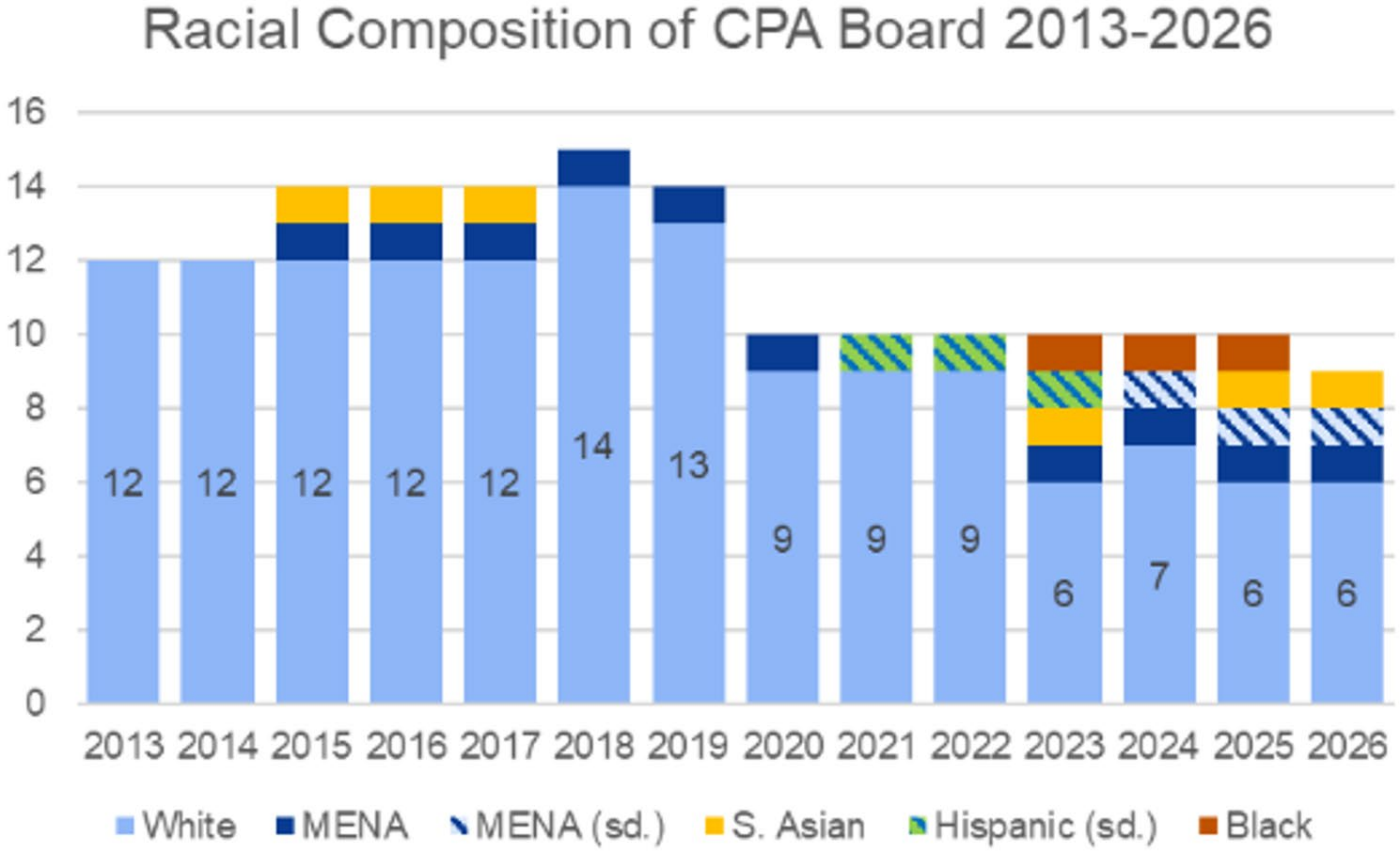
Racial composition of CPA board over a 13-year period. MENA = Middle Eastern, North African, Arab; Stripes = student member (sd). Numbers on the bars indicate the White members

The leadership of the CPA is not representative of Canada’s diversity (Faber, Cenat et al. [21]). The 2021 census recorded 69.4% of the population as White, with increasing numbers of visible minorities and Indigenous people [31]. The CPA leadership positions of CEO, Deputy CEO, and Executive Director, which can be held for as long as those in power desire, have been held by a White person every year, since 1994. Board of Directors data from 2013 to present shows that BIPOC inclusion has been low or absent, and often been achieved through the diversity of student representatives. On average, over the last 13 years, the Board has been 87.1% White excluding student members, as shown in Fig. 1.

### Theoretical framework

This study draws on scholarship examining organizational governance, power hoarding, policy weaponization, and organizational control within professional organizations [23, 24, 26, 27].

The governance changes examined in this case closely align with the concept of power hoarding, which is the accumulation and maintenance of control within a restricted group at the expense of transparency and accountability [37, 40].

Governance scholars have long recognized the importance of limiting the concentration of organizational authority through appropriate oversight and accountability mechanisms. Early work on corporate governance emphasized the separation of ownership and managerial control (Berle & Means [7]), while later agency theory and corporate governance scholarship examined how governance structures can reduce agency problems and constrain managerial power [32, 42]. Similar concerns regarding the distribution of authority, accountability, and oversight have also been examined in nonprofit organizations [10, 15].

Consistent with Lurie and Frankel [35], this study conceptualizes organizational governance through an analogous separation of legislative (Board/Bylaws), executive (Management/CEO), and judicial (Ethics/Oversight/Sections) functions. Within this framework, governance reforms can be evaluated according to whether they distribute authority across these organizational functions or concentrate decision-making within a smaller number of actors.

This concern with the distribution of authority is consistent with broader organizational scholarship on power. Fleming and Spicer [28] identify power as a fundamental feature of organizational life and distinguish between overt forms of influence and more systemic forms that become embedded in organizational structures and routines. In this view, power is not limited to individual decisions but is also exercised through institutional arrangements that shape who participates in governance, whose voices are prioritized, and which organizational outcomes become possible.

Furthermore, individuals occupying high positions of institutional power frequently experience a sense of threat in response to diversity related messages [34]. Prior scholarship suggests that initiatives aimed at redistributing power or addressing systemic inequities often encounter institutional and individual resistance [50]. This resistance may manifest through formal governance mechanisms, policy revisions, or administrative restructuring rather than overt opposition [26].

When these formal mechanisms are misused, it results in the weaponization of policy, which describes the strategic use of seemingly neutral governance rules to secure power and advantage. This mechanism deploys policies that maintain an outward appearance of egalitarianism, yet are designed or applied to yield racially discriminatory or suppressive outcomes [23, 39].

## Method

### Design

This study employed a qualitative case study approach using document analysis to examine proposed governance changes within the CPA and their potential implications for organizational power distribution, representation, and section autonomy. Case study research is well suited to investigating contemporary organizational phenomena within their real-world context, particularly when organizational processes, decision-making structures, and institutional dynamics are central to the inquiry [9, 18]. Documentary evidence served as the primary data source for examining how proposed governance reforms may reshape authority, representation, and organizational functioning within the Association.

### Data sources

The analysis drew upon publicly available organizational documents, including: (a) the CPA Strategic Plan 2020–2025 (CPA [11]); (b) the CPA bylaws in effect prior to the proposed amendments, as well as the proposed bylaw amendments circulated to CPA members in 2025 (Supplementary Materials 3 and 4); (d) publicly available communications describing or explaining the proposed governance changes (Supplementary Materials 1 and 2); and (e) published and publicly available commentary regarding representation, governance, and equity within the CPA (e.g., Williams [48]).

These documents were selected because they directly addressed the governance structure of the organization, the proposed amendments, and the stated strategic commitments relevant to equity diversity and inclusion.

Several authors were present at member information sessions and governance discussions related to the proposed amendments. Observations from these events informed the authors’ understanding of the organizational context but were used only as background information and not subjected to formal analysis relevant to equity, diversity, and inclusion.

### Analytic approach

The authors conducted a qualitative document analysis focused on identifying changes in the allocation of authority, decision-making powers, oversight mechanisms, and Section autonomy. Document analysis is a recognized qualitative method that allows researchers to examine organizational texts as expressions of institutional priorities, governance practices, and power relations [8, 9, 14].

The analysis proceeded through iterative reading and comparison of the original and proposed bylaw language. Particular attention was paid to provisions that altered relationships between the Board of Directors, the Chief Executive Officer, and CPA Sections. Proposed amendments were compared with the corresponding language in previous bylaws to identify substantive changes in authority, accountability, representation, and organizational control.

Interpretations were informed by a power-hoarding framework developed in prior work examining organizational governance, power concentration, and resistance to structural change [26], supplemented by scholarship on hierarchy, empowerment, and organizational control [20, 30]. The analytical tables presented in this paper summarize the authors’ interpretation of the functional implications of specific bylaw amendments and are intended as an interpretive framework rather than a definitive analysis.

### Reflexivity & positionality

The authors acknowledge that they approach this case from positions that include professional, scholarly, and organizational interests in equity, governance, and representation. Several authors have participated in CPA Sections and have engaged in discussions regarding organizational diversity and inclusion. One member is part of the CPA Accreditation Panel. Consistent with qualitative research principles, these perspectives are viewed not as sources of bias to be eliminated but as positions requiring transparency and reflexive consideration throughout the analytic process (APA [2]).

To enhance credibility, interpretations were discussed among all authors and evaluated against the text of the bylaws, strategic planning documents, and relevant governance literature. The purpose of this analysis is not to determine intent on the part of organizational leaders, but rather to examine how the proposed governance changes may reasonably be understood within established theoretical frameworks concerning power, representation, and organizational change.

The authors of this paper are practitioners, educators, and researchers with an interest in mental health equity. The first author is an African American based in Germany, holding a doctorate in biological sciences and an MBA; she possesses an extensive background as a neuroscientist and pharmaceutical expert, focusing on clinical trials and equity initiatives. The second author holds a law degree and is a PhD candidate at the University of Ottawa whose research explores the role of community in psychedelic integration, spirituality, and healing, with a focus on ethics and social inclusion. The third author is a registered psychologist and Canada Research Chair for Mental Health Innovation and Equity, with an extensive publication record in mental health disparities. The fourth author is a Black francophone registered psychologist, tenured professor and research chair, specializing in Black Health; his work centers on racial disparities in mental health, trauma, and systemic racism.

## Findings

The following tables provide a detailed analysis of the most pertinent proposed amendments (all section numbers are based on the organization’s 2025 proposal, as provided in Supplementary Materials 4). Table 1 details changes affecting Section governance and oversight, whereas Table 2 outlines changes representing the consolidation of administrative authority.

**Table 1:** Changes affecting section governance and oversight

Observed governance change	Interpretive governance theme	Proposed bylaw change	Potential organizational implications
**New powers to directly control and discipline constituent sections (sect. 8.03)**	**Mechanism for executive removal and replacement**	Addition: “…the Board may, instead of dissolving a Section, remove and replace the members of the Section executive in the event that the Section breaches any Governance Policies”	Creates a mechanism through which the board may remove and replace elected section leadership in cases of perceived non-compliance with governance policies. This reduces the independence of sections and could affect their ability to challenge or critique Board decisions
**Gag order on public statements (sect. 8.05(c))**	**Restriction on independent public advocacy**	Addition: Clauses forbidding Sections from making “any public statements, issue position papers or policy statements without the express written consent of the Board…”	Removes the ability of Sections to advocate independently on issues relevant to their members (e.g., systemic racism). It centralizes the organization’s voice, requiring BIPOC Sections to secure approval from a predominantly non-BIPOC Board before speaking on DEI issues
**Deletion of powers (sect. 8.09)**	**Formal diminution of section autonomy**	Deletion of the entire section outlining the powers of Sections to “initiate and undertake activities,” “draft position papers,” and “organize meetings”	Strips Sections of their explicit, by-law-enshrined autonomy, transforming them from semi-autonomous bodies into fully subordinate committees dependent on the Board’s administrative discretion

**Table 2:** Consolidation of administrative authority

Observed governance change	Interpretive governance theme	Proposed bylaw change	Potential organizational implications
**Centralization of oversight to CEO (Sect. 8.01)**	**Delegation of rule-making authority**	New clause allowing the Board to “delegate the development and implementation of section operating regulations to the Chief Executive Officer”	Shifts key rule-making power to a single, unelected staff member, circumventing transparent Board processes and limiting the influence of elected Section representatives
**Broadened disciplinary grounds (sect. 3.06(a))**	**Expansion of sanctionable offenses**	Expansion of disciplinary grounds to include violations of “or other policies and regulations of the Association”	Broadens the scope for sanctioning members from formal By-laws to a wider, more easily changed set of executive-created policies, increasing the net for disciplining internal dissent
**Removal of partners from board meetings (sect. 6.09(b))**	**Shielding board activities from scrutiny by peers**	Change where partner representatives were “entitled” to attend Board meetings, to the Board “may invite” them for “portions thereof”	The activities of the Board had some level of oversight and input by peer organizations, but the change allows the Board to function as a secret echo chamber
**Removal of checks on board ethics (sect. 2.07)**	**Reduction of democratic safeguards**	Deletion of the phrase “board code of conduct and conflict of interest” from the definition of governance policies in the by-laws	The Board alone writes, controls, and creates their own code of ethics

Critically, none of the proposed changes expand Section authority or increase power of the organization’s membership (Supplementary Materials 3 and 4).

### Analysis of organizational resistance

There are four power hoarding tactics [26] in particular that are present in this case:

#### Usurpation of judicial function

The proposed addition allowing the Board to remove and replace elected Section executives (Sect.  8.03) is the legislative/executive arm co-opting the judicial/oversight function. The Sections, particularly the BIPOC Sections, act as a vital check and balance for the organization and its membership, voicing concerns, highlighting gaps in clinical research and practice, proposing equitable solutions to those gaps, and advocating for members. The proposed change appears to substantially reduce the independence of this community accountability mechanism and member resource, rendering it subordinate and vulnerable to the very authority it is meant to hold accountable.

#### Reinforcing status quo

The clarification that top leadership positions are at-large (Sect. 5.03(c) preserves the existing structure through which Board leadership is selected and entrenched. Because representation on governing bodies is often shaped by existing networks, nomination processes, and institutional traditions, governance arrangements that maintain existing selection mechanisms may also maintain existing patterns of representation [33, 49]. In the context of ongoing calls for designated BIPOC representation, retaining the existing structure may be understood as prioritizing continuity over structural reform, community representativeness and equitable governance.

#### Reduction of democratic safeguards

A third, and perhaps most blatant tactic of organizational control is the dismantling of democratic safeguards to permanently insulate leadership from accountability [26]. This operates in the CPA through two interlocking mechanisms.

First, CPA governance documents establish a formal quorum of only 25 individuals out of approximately 7,000 members, meaning sweeping changes can be codified by a mere 17 people (0.25% of the membership). When members raised concerns regarding the initial timeline of this specific vote, the administration adjusted the process by extending the voting window. Ultimately, the total engagement remained highly localized: the final tally recorded 111 votes in favor, 41 opposed, and 13 abstentions. While fully compliant with formal procedures, this collective turnout represents just 2.36% of the total association membership, demonstrating how sweeping governance overhauls can be codified by a tiny fraction of the constituency.

The deeper systemic issue, however, lies in how this low-quorum vote was weaponized to shift the balance of organizational power to the executive. By deleting explicit Section autonomy (Sect.  8.09) while simultaneously removing the “Board code of conduct and conflict of interest” from the strict oversight of the formal By-laws (Table 2), the Board effectively granted itself the unvetted authority to self-define its ethical parameters. This is a striking inversion of basic governance principles. These new Governance Policies and operating regulations can be written, altered, and enforced solely by the Board or the CEO (Sect.  8.01) without ever requiring another membership vote. Thus at the very moment that Sections are subjected to an erosion of their rights and responsibilities, the Board becomes less accountable to the governance safeguards designed to oversee it. The asymmetry is particularly notable given the Board’s expanded authority over Section Governance, including the power to remove and replace elected Section executives (Sect.  8.03) which sits at the heart of concerns raised by the proposed reforms. From a governance perspective, this is a remarkable and troubling combination.

#### Controlling the room

Control of organizational discourse represents a particularly effective form of power consolidation because it shapes not only who may speak, but also which concerns become visible to the broader membership. By requiring Board approval for public statements, position papers, and policy communications, authority over organizational messaging becomes increasingly centralized. Such arrangements limit the capacity of constituent groups to independently raise concerns, propose solutions, advocate for their communities, or challenge prevailing organizational priorities. Over time, control of communication can influence which issues receive attention, which concerns are normalized, and which perspectives remain largely absent from organizational discourse.

When paired with communication strategies that minimize the above structural overhauls as “primarily administrative,” these tactics serve as powerful mechanisms to covertly strip Section autonomy, erode good governance and block equity initiatives.

Furthermore, a recurring theme across the proposed amendments is the distinction between procedural and structural change. Procedural modifications alter how decisions are made, communicated, or implemented, whereas structural changes alter the distribution of authority itself. Organizational scholars have noted that hierarchy often persists even when organizations adopt reforms that appear to decentralize authority. Rather than eliminating hierarchy, structural changes may alter the balance between formal and informal forms of control while preserving underlying power relationships [20]. Similarly, Hardy and Leiba-O’Sullivan [30] argue that organizational empowerment initiatives often devolve selected responsibilities while preserving control over key resources, decision-making processes, and organizational priorities.

While procedural reforms may improve administrative efficiency, they do not necessarily increase representation, accountability, or shared governance. Evaluating governance reforms therefore requires attention not only to administrative function, but also to whether authority becomes more broadly distributed or increasingly concentrated within existing leadership structures. These amendments described systematically shift authority away from Sections and toward the Board and CEO. Because the proposals emerged in the context of explicit demands for greater BIPOC representation, and because the affected Sections were among the primary vehicles for such advocacy, the reforms can reasonably be understood as organizational resistance to structural diversity.

### The rhetoric of resistance: strategic framing and JEDI backlash

The communication strategy employed by the CPA leadership (i.e., the choice of wording used in their email, online posts, and townhall meetings; Supplementary Materials 1 and 2) illustrates how organizational change can be framed to strategically minimize scrutiny and manage internal dissent.

The leadership’s decision to characterize a major power shift as merely “administrative” is consistent with patterns of diversion and denial, described in the organizational change literature [1, 26, 43, 44] which attempt to bypass scrutiny by labeling the changes as non-political. In this case the denial is that the leadership disavows the political nature of the amendments (e.g., denying that the ability to remove elected leaders is a tactic to hoard power). The diversion is manifested in how the focus is diverted to issues of “administrative best practice” and “efficiency,” preventing members from scrutinizing the core impact on Section autonomy and representation.

The present case also illustrates a broader phenomenon that has been described as institutional deflection [41]. Rather than openly contesting demands for structural reform, organizations may redefine the scope of the issue in ways that narrow perceived responsibility or minimize the significance of proposed changes. In the present case, governance amendments with potentially substantial implications for Section autonomy, representation, and organizational accountability were presented primarily as administrative updates. Such framing does not necessarily invalidate the amendments themselves, but it may influence how members understand their significance and thereby the degree of scrutiny applied during the decision-making process.

This strategic framing, combined with the low quorum threshold, has the effect of reducing opportunities for broader member deliberation regarding the proposed governance changes, effectively bypassing the larger member base to enact the power consolidation.

## Discussion of structural dynamics

### Structural racism and weaponization of policy

The operation of systemic racism in institutions and organizations is a well established finding [3, 51]. The actions of CPA leadership align with critical race theory, specifically the tenet that structural improvements for marginalized groups are often permitted only when they do not fundamentally challenge the existing racial hierarchy [5, 19].

The functional autonomy of identity-focused Sections may challenge White-dominated leadership, as these groups bring visibility to issues previously ignored. Real change requires sharing of power, and the entirely predictable resistance to sharing power often undermines the intended effectiveness of diversity-focused subcommittees. As Fleming and Spicer [28] note, power is often exercised through organizational arrangements that determine who participates in governance and whose perspectives shape institutional priorities. Policy tools are the weapons which are being utilized to thwart power sharing initiatives and weaponization of policy is the method by which disempowerment occurs [26]. Such resistance is consistent with broader organizational scholarship suggesting that institutions often respond to demands for greater inclusion by creating mechanisms for participation that leave core authority structures unchanged [20, 30]. This is a call to organizations at large, to be aware of these dynamics and implement systematic processes to prevent authoritarian power concentration and ensure decision-making is fairly distributed.

The distinction between participation and authority is particularly relevant when considering representation within membership-based professional organizations. Consultation mechanisms, advisory bodies, and opportunities for input may increase participation, but they do not necessarily alter decision-making power. Representation becomes structurally meaningful only when affected groups possess the authority to influence outcomes rather than merely provide feedback (e.g., [23]). Organizational hierarchy is defined not merely by opportunities for participation but by the allocation of authority and influence within decision-making structures [20]. Participation alone does not necessarily redistribute power unless stakeholders gain meaningful access to decision-making processes and organizational resources [30]. Consequently, governance analyses must distinguish between opportunities to be heard and opportunities to exercise institutional power.

### Advantages of quantifying power - practical implications

Although it is well understood that power must be divided to avoid dictatorships and provide checks and balances, the precise division and categorization of tasks which are permitted to be carried out by each governing body is not always clear. This is why it is important to explicitly consider power inherent in decision-making tasks which are enshrined in the bylaws, in key moments and whenever these are suggested to be changed [26, 29].

Effective governance requires the explicit quantification and allocation of power via documented frameworks, such as detailed ‘delegation of authority’ protocols [46]. This transparency prevents the accumulation of unchecked power. These practices help to promote transparency, reduce corruption and ensure that power is not concentrated in a single unit, ultimately enhancing corporate governance. Improper definition and allocation of tasks to their respective roles, or a lack thereof, can lead to role ambiguity and unequal power dynamics which can hinder organizational effectiveness [29]. Furthermore, best practices in well-managed Boards make it clear that Board members, not only the executive leadership team, must possess and exercise executive or decision-making powers [46].

Conversely, dysfunctional organizations often view power as a finite commodity, leading leaders to hoard control rather than distribute it [26]. This fear-based scarcity mindset devalues power-sharing and fosters resistance to transparency [6]. The psychological reason for holding on to power so dearly is rooted in fear, which is linked to the notion that there can never be enough power to keep the organization and its beneficiaries “safe” [6, 26, 27, 40]. A lack of transparency and ill-defined tasks hides covert capture of power by executive level entities as ultimately, power is that much more powerful when it operates from the shadows.

### Disempowerment of JEDIs - practical implications

Although Justice, Equity, Diversity and Inclusion committees (JEDIs) on Boards of Directors have been established in recent years, they have often been unable to achieve their intended goals [16]. The reasons for these failures can be summarized by a lack of power. The governance changes examined in this case raise concerns that identity-focused sections may face challenges similar to those described in the literature on structurally disempowered JEDI bodies. They are typically granted limited binding authority or resources, leaving them vulnerable to executive override and unable to enact necessary structural changes [16].

In order for JEDI initiatives to succeed, equity and diversity initiatives must be prioritized as strategic goals endorsed by the Board and senior executive leadership. The culture of an organization is heavily influenced by the leadership, and if the leadership is not fully committed, mandates will not permeate throughout the organization. Leader and member buy-in correlates with the success of JEDI initiatives, and the failure of such structures reflects an unwillingness to make necessary changes to structure and policies, rather than inherent flaws in the structure itself [13].

### Public health implications

The governance issues examined in this case have implications that extend beyond the internal functioning of a professional association. Professional organizations help shape the culture, priorities, and future composition of their respective fields. When organizations resist efforts to increase structural representation, equity, accountability and shared governance, they may contribute to nurturing contexts that are experienced as unwelcoming or exclusionary by members from historically marginalized groups [45]. Such environments can discourage recruitment, retention, advancement, and leadership participation among Black, Indigenous, and People of Color (BIPOC) psychologists, thereby reinforcing longstanding patterns of underrepresentation within the profession [22].

These concerns have broader public health implications. Canada’s population is becoming increasingly diverse, yet the psychology workforce continues to inadequately reflect this diversity [4, 47]. A lack of representation within professional psychology limits the availability of culturally responsive services, reduces trust in mental health systems among underserved communities, and contributes to persistent disparities in access to care and treatment outcomes. Consequently, governance decisions that affect representation, inclusion, and power sharing within professional organizations should not be viewed solely as internal administrative matters. Rather, they may influence the profession’s capacity to recruit and support a diverse workforce capable of meeting the mental health needs of Canada’s increasingly multicultural population.

### Proposed expansion of section powers

To counteract the executive centralization documented in this case, the CPA’s By-Laws should be amended to further empower Sections and amplify their voices. This will also enable these sections to function as essential institutional checks on the Board. Specifically, we propose two key amendments that expand Section authority and are conceptually aligned with recent scholarship on this issue [26]. First, Sections should be granted Veto Power over any proposed bylaw or policy change that directly or disproportionately impacts a marginalized group represented by that Section (Faber, Cenat et al. [21]). This power ensures that attempts to suppress dissent or roll back JEDI initiatives (as seen in Sect. 8.03 and 8.05(c) cannot proceed without the consent of the affected membership.

Second, Sections should be granted Initiation Power, allowing them to bring forward non-binding resolutions to the general membership for a vote, even without Board approval. This restores the explicit power to “initiate and undertake activities” that were previously deleted (Sect. 8.09), guaranteeing a formal democratic pathway for internal advocacy. Institutionalizing these structural powers is essential to prevent the covert weaponization of policy by any executive body.

Finally, sections must retain the right to draft position papers on topics of relevance to the Section, to initiate public policy statements in areas of expertise, and to organize meetings without undue interference from the Board. The role of the Board is to support these activities and not suppress them and facilitate their execution in a timely manner.

### Recommendations

Rules are the basis for norms, on which the culture is built. In well-run Boards, charters are developed that define the decision-making powers for Board committees, as ad hoc decision-making is antithetical to the success of JEDIs [26, 46]. Best practices suggest empowering such bodies with clear authority to circumvent disempowerment by policy weaponization, with the understanding that even if these measures are not necessary at the outset, the nature of systemically racially monolithic structures will work to create inequity over time [29, 46]. Many of the proposed amendments to the bylaws under Sect. 8 functionally remove the ability of these groups to self-govern and give the Board sweeping powers to remove members or dissolve the section entirely if it is deemed to ‘breach any governance policies.’ This heavy-handed oversight without any indication of reporting mechanism, due process or Board accountability connected to this decision-making power goes against everything the CPA has purported to strategically prioritize in its own 2020–2025 Strategic Plan. Broad and sweeping powers are not in service to an equitable and accountable governance culture, and JEDIs should have input and veto power over all organizational decisions that impact marginalized groups.

#### Diversification of the board

The most direct and actionable recommendation to safeguard structural equity is the mandatory diversification of the Board of Directors ([24]; Faber, Cenat et al. [21]).

The Government of Canada has formally recognized the benefits of having a Board-level diversity policy as one of the most significant steps to promote equity. They recommend a written policy relating to the representation of women, Indigenous Peoples (First Nations, Inuit and Métis), racialized people and people with disabilities on the board of directors and among senior management (Corporations Canada [17]). A written policy allows assessment of the corporation’s current diversity and provides a basis for discussion on ways to improve diversity. As part of a recent initiative called the 50–30 Challenge, organizations are tasked with at least 50% female leadership and 30% membership for those in equity-deserving groups. Corporation Canada underscores the benefits of having a more diverse organization, complete with a toolbox of resources available to assist organizations in continuing their efforts toward diverse and inclusive workplaces.

As we have underscored, this goal can be achieved at the CPA through an amendment to bylaw 5.03 about the composition of the Board, where the CPA can leverage the opportunity to establish designated seats not just for Sections but specifically for BIPOC members, which would serve as a permanent check against the power hoarding observed in this case. This low-hanging fruit would not only provide meaningful progress towards CPAs strategic priority for modeling inclusion but would also ensure ongoing structural engagement and stewardship of equity priorities, and the need to ensure CPA continues to improve its accountability towards and service of BIPOC members and Canadian citizens.

With this improved board composition, there would also be an opportunity to adjust bylaws 5.04 and 5.05, or temporarily leverage bylaw 5.06, to potentially allow these positions to be strategically filled by the elected leaders of the constituent CPA Sections focused on the interests of BIPOC members. Appointing Section leaders in this capacity ensures that representation is not merely symbolic, and rather is chosen by the community they represent, and is anchored by organizational expertise, a membership mandate, democratic processes, and direct accountability to the communities demanding and cultivating structural equity and accountability. These changes have the potential to transform CPAs governance approach from a mechanism of resistance into one of genuine reform and strategic relevance (Faber, Cenat et al. [21]).

#### Outside consultation

While the specific issues addressed here are pressing, they are symptomatic of deeper, systemic challenges that actively undermine the organization’s ability to realize the goals outlined in its own strategic plan. Therefore, we recommend all organizations with these challenges engage an independent diversity and equity consultant to conduct a comprehensive evaluation of the issues. True democratic governance requires the capacity to think flexibly, receive open input, and engage in difficult conversations that are essential for meaningful progress. An external expert can facilitate this necessary process.

## Conclusion

Building on prior scholarship examining organizational governance, power concentration, and resistance to structural change [25, 30], this case study demonstrates how seemingly ‘administrative’ governance reforms may significantly alter power relationships within professional organizations. These findings are consistent with broader organizational scholarship suggesting that hierarchical relationships often persist across diverse organizational forms, including those that emphasize participation, decentralization, or collaborative governance [20].

The CPA case illustrates that resistance to structural equity is rarely overt. Instead, it is expressed through systemic governance restructuring, specifically, by creating explicit mechanisms that silence dissent and institutionalize the maintenance of pre-existing power distributions. The specific amendments analyzed here created explicit mechanisms to silence dissent and remove sectional power and elected leaders, providing the central authority with tools to suppress structural calls for equity.

This study examines the mechanisms of an organizational power struggle and reveals inequity and biased practices. The findings contribute to emerging discussions regarding the interaction between governance structures, representation, and organizational equity initiatives. The case study, which elucidates how power hoarding may be implemented, can serve as a valuable resource for consultants and organizational psychologists. To prevent such subversion within professional bodies, it is essential to institutionalize divided power, codify representation, and include robust checks and balances [25, 29, 46].

## Supplementary Information

Below is the link to the electronic supplementary material.

Supplementary Material 1: Notice of Special Meeting of CPA Members. This document is the email official notice distributed to CPA members announcing the December 11, 2025 special meeting convened to present and vote on the proposed bylaw revisions. It is included because it establishes the formal context, timeline, and rationale provided to members regarding the governance changes under examination in this study.

Supplementary Material 2: Annual General Meetings and Reports – CPA. This document, posted on the CPA website, provides the CPA Board’s public explanation and justification for the proposed bylaw revisions, including descriptions of specific amendments and their stated purposes. It is included because it offers an official organizational interpretation of the proposed governance changes and serves as a key source for understanding how the revisions were presented to members.

Supplementary Material 3: CPA Draft By-Laws, October 2025. This document contains the original red-line version of the proposed bylaw revisions distributed to CPA members on October 29, 2025. It is included because it serves as a primary source document for identifying and analyzing the proposed governance changes examined in the case study.

Supplementary Material 4: CPA Draft By-Laws, November 2025. This document contains the revised red-line version of the proposed bylaw amendments posted on November 4, 2025, after CPA identified omissions in the originally posted version. It represents the most complete record of the proposed revisions and allows comparison between versions to ensure accurate analysis of the governance changes under consideration.

## Data Availability

All datasets generated or analysed during the current study are available from the first author by request.
