# Supplementary material for: CPA governance restructuring and section autonomy: a case study in organizational resistance to structural diversity
Source: Int J Ment Health Syst. 2026 Jul 30;20:26. doi: 10.1186/s13033-026-00722-z (PMC13459331; doi:10.1186/s13033-026-00722-z)
Supplement: Supplementary file 1 [file 13033_2026_722_MOESM1_ESM.pdf]

Subject: Notice of Special Meeting of CPA Members
Date: Wednesday, October 29, 2025 at 3:27:49 PM Eastern Daylight Saving Time
From: CPA Membership

Governance

A special meeting of members of the Canadian Psychological Association will be held virtually, on Thursday December 11, 2025, at 1 pm Eastern Standard (EST) for the purposes of presenting and voting on proposed revisions to the By-laws of CPA that have been approved by the CPA's Board of Directors.

The proposed revisions to the By-laws are as noted in the red-line version which can be found by clicking here:
https://cpa.ca/docs/File/Governance/CPA%20-%20Draft%20By-Laws%20-%20October%202025_Final_EN.pdf

We encourage all CPA members to review the proposed revisions. The proposed By-law revisions are administrative in nature to help the CPA best meet the needs of its membership and achieve best practice in governance in compliance with Canada's Not-for-Profit Corporations Act (CNCA).

On Thursday November 13th at 1pm (EST) we will convene a virtual Town Hall during which members can put forth any questions about the proposed revisions to the CPA's President, Dr. Steven Smith and the CPA's CEO, Dr. Lisa Votta-Bleeker.

The proposed revisions to the CPA's By-laws will be put forward for approval pursuant to the following special resolution of the members: BE IT RESOLVED that the proposed revisions to the CPA By-laws, as reflected in the notice of the meeting, be approved.

A special resolution needs to be approved by 2/3 of the membership present to vote. Quorum is defined as 25 members. Voting will be open to all members of the CPA and must occur from 1:00 – 2:00pm EST on December 11th, 2025.

This is an important opportunity for the membership to participate in a process that ensures best practice in governance for the Association. Your understanding of the proposed revisions, and your participation in the change process, is important.

Instructions on how to attend the virtual members meeting by webinar, and to exercise your vote electronically, will follow in a separate email.

Should you have questions about the meeting or Town Hall, please contact us at governance@cpa.ca

BY ORDER OF THE BOARD OF DIRECTORS, on the 29th of October 2025.

Lisa Votta-Bleeker, Ph.D.
Chief Executive Officer
Canadian Psychological Association

BMS

BMS is the official Liability Insurance Provider for members of the CPA.

Members of the Canadian Psychological Association have access to an industry leading liability insurance program, brokered by BMS Canada Risk Services Ltd (BMS Group). More...

CANADIAN PSYCHOLOGICAL ASSOCIATION

SOCIÉTÉ CANADIENNE DE PSYCHOLOGIE

To ensure that you continue to receive your emails from the Canadian Psychological Association, please add our email address (example: email@cpa.ca) to your Address Book or Safe Contacts List. Thank you.

To stop receiving these emails Click here to un-subscribe.

Canadian Psychological Association
1101 Prince of Wales Drive, # 230 Ottawa, ON K2C 3W7
(tel) 613-237-2144
(toll free in Canada) 1-888-472-0657
