# Supplementary material for: CPA governance restructuring and section autonomy: a case study in organizational resistance to structural diversity
Source: Int J Ment Health Syst. 2026 Jul 30;20:26. doi: 10.1186/s13033-026-00722-z (PMC13459331; doi:10.1186/s13033-026-00722-z)
Supplement: Supplementary file 2 [file 13033_2026_722_MOESM2_ESM.pdf]

CANADIAN PSYCHOLOGICAL ASSOCIATION
SOCIÉTÉ CANADIENNE DE PSYCHOLOGIE

Annual General Meetings and Reports

By-Law Revision Explanation

A review of By-laws every two to five years is recommended to ensure that By-laws are current and accurately reflect the organization's operations and comply with applicable laws and regulations. The CPA last reviewed its By-laws in 2018. In 2023, the CPA undertook a review of its Governance Policies and Operating Regulations; these were updated and approved by the CPA's Board in June 2024. Following this, the CPA's legal counsel reviewed the CPA's By-laws to ensure alignment and compliance between all documents. Recommended revisions to the By-laws were presented to the CPA's Governance Committee, which has oversight of the CPA's By-laws, rules, and procedures. The Governance Committee presented the proposed By-law revisions to the Board at its June 2025 meeting; proposed administrative changes were approved. At its October 2025 Board meeting, additional discussion of the By-laws took place and the process for bringing forward the proposed revisions to the membership for approval was finalized.

On Wednesday October 29th, the CPA sent notice to the membership of a special meeting to approve proposed revisions to the By-laws. This special meeting would be preceded by a Town Hall on November 13th during which the membership could ask any questions and obtain more information on the proposed By-laws.

Since this communication, questions have come forward from the membership regarding the proposed revisions, and I would like to take this opportunity to express our appreciation to those who have taken the time to engage and seek clarification. Your thoughtful questions have highlighted the need to provide you with more information about the rationale for the revisions in advance of the Town Hall.

Note that two versions of the approved red-line revisions are now posted on the website. In preparing this document, we realized that not all the approved red-line revisions were showing in the first posted document.

- The two "whereas" statements were removed as they were no longer

necessary as per our legal counsel.

- Some of the changes involve a change of wording (e.g., use of the term Association in place of Corporation, Governance Policies in place of Operating Regulations), others involve capitalizing words that were previously noted in lower-case, and others were re-written for clarity (e.g., 1.02-d).
- Section 2.03 (Public Accountant) – Re-written to more accurately reflect the process by which the Members appoint the CPA's public accountant. The original provision set out requirements for the members to resolve not to appoint a public accountant, which are not relevant because the CPA will always seek to appoint a public accountant.
- Section 2.08 (Affiliates) – Honorary Fellows was removed from the list as they are not Affiliates.
- Section 4.07 (Chair of the Meeting) – Updated to indicate that the incoming President or Immediate Past President would serve as Chair of a meeting of the Members if the Chair is unavailable to chair a meeting.
- Section 4.15 (Resolution in Lieu of Meeting) – Removed as would never be feasible to get signatures of all members entitled to vote so would never have a resolution in lieu of meeting.
- Section 5.03 (Composition of Board) – Clarification was added regarding the five Directors-at-large on the Board to note that the President and Immediate Past President are considered Directors-at-large.
- Section 5.05 (Absentee Voting by Mailed-In Ballot or Electronic Ballot) – Removed to align with current voting procedures.
- Section 5.06 (Nomination of Directors) – Updated to reflect current nominating procedures and role of the Nominating Committee.
- Section 6.08 (Board Committees and Advisory Bodies) – The names of the Board Committees were removed such that a Board Committee could be established and sunset in the future without requiring a By-law change.
- Section 7.02-b (President) – Updated to align with description of role as per current Governance Policies.
- Section 8.01 (Sections – Purpose) – As written, stated that Governance Policies regarding Sections fall under the purview of the Board; added that the development and implementation of Section Operating Regulations could be delegated to the CEO.
- Section 8.03 (Dissolution and Restructuring) – As written, Section 8.03 did not fully articulate all the situations under which a Section could be dissolved or restructured by the Board. As previously written, the only instances when a Section could be dissolved were if the Section voted/requested dissolution or if a Section fell below the

required number of 25 members to maintain Section status. However, within the Governance Policies, there are other instances under which a Section could be dissolved or restructured (such as when a Section Chair cannot be found or a Section asks to merge with another Section), and the proposed revision was meant to address that. There are also instances in which a Section Executive could be asked to step down – these include: mismanagement of Section funds, violating the CPA's Code of Conduct, violating a CPA By-law (e.g., opening a line of credit), and failing to fulfill the responsibilities of Section Executive as outlined in the Section Operating Regulations. These tools are meant to protect the CPA. Dissolving a section or removing a member of a Section executive for a breach of the Governance Policies would require the approval of the Board, who must act in the best interests of the CPA and be duly diligent.

- Section 8.04 (Organization) – Removed all the specific items that were noted as requirements in the Section Terms of Reference, such that the Model Terms of Reference requirements could be specified in the Section Operating Regulations thereby allowing for greater ease of change to the Model Terms of Reference.
- Section 8.05 (Regulation of Section Affairs) – The proposed revision is meant to more fully capture the range of activities in which a Section can engage and note the circumstances under which approval of a given activity is to be sought. In addition, rather than have separate provisions governing the powers of Sections, we are proposing to combine Section 8.05 and Section 8.09. As such, we have added the contents of Section 8.09 regarding public statements, etc. to Section 8.05, and then removed Section 8.09.
- Section 8.07 (Reports) – Revised to more accurately reflect when an update from the Section is required on its activities for inclusion in the Annual Report (8 weeks rather than 4). It was also revised to indicate what is not required in the report (i.e., budget for the ensuing year).
- Section 8.09 (Powers) – This Section is being proposed to be removed as it was deemed by the CPA's legal counsel to be redundant given the proposed edits to Section 8.05 re: Regulation of Section Affairs.
- Section 9.01 (Method of Giving Notice) – This Section was revised to remove reference to a Secretary as the CPA does not have a Secretary.
- Section 10.02 (By-laws to be confirmed by Special Resolution) – removed because it was redundant given the proposed edits to other sections.
- Section 10.02 (Effective Date of Board initiated By-law, Amendment or Repeal) lowered the threshold to amend the By-laws from a special resolution to an ordinary resolution.

- Section 10.04 (Repeal of By-laws) – Clarification was added to this By-law to include Directors and Committee members in the list of volunteers to act as appointed (elected was also added) in the case of a By-law repeal.

I hope this addresses some of the initial questions that the membership may have. I look forward to addressing any other questions during the Town Hall and encourage all members to attend.

Sincerely,
Steven Smith, Ph.D.
CPA President, 2025-2026
On behalf of the CPA's Board of Directors

Proposed By-Laws:

Second version with all red-line changes appearing posted Nov 4, 2025: https://cpa.ca/docs/File/Governance/CPA – Draft By-laws_2025-66445218-v4 – November 2025.pdf

Original version posted Oct 29, 2025: https://cpa.ca/docs/File/Governance/CPA%20-%20Draft%20By-Laws%20-%20October%202025_Final_EN.pdf

---

Notice of Special Meeting of CPA Members

A special meeting of members of the Canadian Psychological Association will be held virtually, on Thursday December 11, 2025, at 1 pm Eastern Standard (EST) for the purposes of presenting and voting on proposed revisions to the By-laws of CPA that have been approved by the CPA's Board of Directors.

The proposed revisions to the By-laws are as noted in the red-line version which can be found by clicking here: CPA – Draft By-Laws – October 2025_Final_EN.pdf.

We encourage all CPA members to review the proposed revisions. The proposed By-law revisions are administrative in nature to help the CPA best meet the needs of its membership and achieve best practice in governance in compliance with Canada's Not-for-Profit Corporations Act (CNCA).

On Thursday November 13th at 1pm (EST) we will convene a virtual Town Hall during which members can put forth any questions about the proposed revisions to the CPA's President, Dr. Steven Smith and the CPA's CEO, Dr. Lisa Votta-Bleeker.

The proposed revisions to the CPA's By-laws will be put forward for approval pursuant to the following special resolution of the members: BE IT RESOLVED that the proposed revisions to the CPA By-laws, as reflected in the notice of the meeting, be approved.

A special resolution needs to be approved by 2/3 of the membership present to vote. Quorum is defined as 25 members. Voting will be open to all members of the CPA and must occur from 1:00 – 2:00pm EST on December 11th, 2025.

This is an important opportunity for the membership to participate in a process that ensures best practice in governance for the Association. Your understanding of the proposed revisions, and your participation in the change process, is important.

Instructions on how to attend the virtual members meeting by webinar, and to exercise your vote electronically, will follow in a separate email.

Should you have questions about the meeting or Town Hall, please contact us at governance@cpa.ca .

BY ORDER OF THE BOARD OF DIRECTORS, on the 29th of October 2025.

Lisa Votta-Bleeker, Ph.D.
Chief Executive Officer
Canadian Psychological Association

2025 Annual General Meeting

- 2025 AGM Agenda
- 2024 AGM Minutes
- 2024-2025 Annual Report

Annual General Meeting Reports can be found in the Members Portal

Elections

Board of Directors

Past CPA Officers & Meetings

About CPA Privacy Site Navigation Contact CPA Terms of Use Sitemap English Français
