# Supplementary material for: CPA governance restructuring and section autonomy: a case study in organizational resistance to structural diversity
Source: Int J Ment Health Syst. 2026 Jul 30;20:26. doi: 10.1186/s13033-026-00722-z (PMC13459331; doi:10.1186/s13033-026-00722-z)
Supplement: Supplementary file 3 [file 13033_2026_722_MOESM3_ESM.pdf]

CANADIAN PSYCHOLOGICAL ASSOCIATION
SOCIÉTÉ CANADIENNE DE PSYCHOLOGIE

Amended: [•], 2025

BY-LAW NO. 1

A By-law relating generally to the conduct of the affairs of

CANADIAN PSYCHOLOGICAL ASSOCIATION
SOCIÉTÉ CANADIENNE DE PSYCHOLOGIE

(the "~~Corporation~~Association")

TABLE OF CONTENTS

Section 1 - Interpretation
Section 2 - Financial and Other Matters
Section 3 - Membership
Section 4 - Meetings of Members
Section 5 - Directors
Section 6 - Meetings of Directors
Section 7 - Officers
Section 8 - Sections
Section 9 - Notices
Section 10 - By-laws

NOW THEREFORE BE IT ENACTED as a general operating By-law of the ~~Corporation~~Association as follows:

SECTION 1 - INTERPRETATION

1.01 Definitions

In this By-law, unless the context otherwise requires:

(a) "**Act**" means the *Canada Not-for-profit Corporations Act* S.C. 2009, c.23 including the Regulations made pursuant to the Act, and any statute or regulations that may be substituted, as amended from time to time;

(b) "**Articles**" means the original or restated articles of incorporation or articles of amendment, amalgamation, continuance, reorganization, arrangement or revival of the ~~Corporation~~Association;

(c) "**Board**" means the Board of Directors of the ~~Corporation~~Association and "**Director**" means a member of the Board;

(d) "**By-laws**" means this By-law No. 1 and any other by-laws of the ~~Corporation~~Association as amended and which are, from time to time, in force and effect;

(e) “**Fellows**” means Members who have made a distinguished contribution to the advancement of the science or profession of psychology or who have given exceptional service to their national or provincial associations, have met the requirements set forth in Governance Policies, who have been recommended as Fellows by the Committee on Fellows and Awards and who have been approved as such by the Board;

(f) “**Governance Policies**” means the ~~operating regulations~~governance policies approved by the Board in accordance with section 2.07 of this By-law;

(g) “**Member**” means any person who has been admitted to membership in the ~~Corporation~~Association in accordance with the By-laws;

(h) “**Ordinary Resolution**” means a resolution passed by a majority of ~~n~~ the votes cast on that resolution;

(i) “**Proposal**” has the meaning given in section 4.14 of this By-law;

(j) “**Regulations**” means the regulations made under the Act, as amended, restated or in effect from time to time;

(k) “**Section**” means a section of the ~~Corporation~~Association, as ~~has the meaning set out~~described in section 8 of this By-law; and

(l) “**Special Resolution**” means a resolution passed by a majority of not less than two-thirds (2/3) of the votes cast on that resolution.

1.02 Interpretation

The following rules of interpretation will apply to this By-law (unless the context otherwise requires):

(a) words importing the singular number only will include the plural and *vice versa*;

(b) the word "person" will include an individual, sole proprietorship, partnership, unincorporated association, body corporate, and a natural person;

(c) words referring to a gender include all genders; and

(d) if any of the provisions contained in the By-laws are inconsistent with those contained in the Articles or the Act, the provisions contained in the Articles or the Act, as the case may be, shall prevail.

SECTION 2 - FINANCIAL AND OTHER MATTERS

2.01 Financial Year

Unless otherwise changed by resolution of the Board, the financial year end of the ~~Corporation~~Association shall be the 31st day of December in each year.

2.02 Banking Arrangements

The banking business of the ~~Corporation~~Association shall be transacted at such bank, trust company or other firm or corporation carrying on a banking business in Canada or elsewhere as

the Board may designate, appoint or authorize from time to time by resolution. The banking business or any part of it shall be transacted by an officer or officers of the ~~Corporation~~Association and/or other persons as the Board may by resolution from time to time designate, direct or authorize.

2.03 **Public Accountant**

At each annual meeting, the Members shall appoint a public accountant to hold office until the close of the next annual meeting and, if an appointment is not so made, the public accountant in office will continue in office until a successor is appointed. The Members may, at any special meeting, remove the public accountant before the expiration of such public accountant's term of office, and shall, by a majority of the votes cast at that meeting, appoint another public accountant in such public accountant's place for the remainder of such public accountant's term. If the Members fail to appoint a successor public accountant, the Directors shall immediately fill any vacancy in the office of public accountant. The remuneration of the public accountant shall be fixed by the Board.

2.04 **Annual Financial Statements**

The ~~Corporation~~Association shall send copies of the annual financial statements and other documents referred to in subsection 172(1) of the Act to the Members between 21 to 60 days before the day on which an annual meeting of Members is held, unless a Member declines to receive them. Alternatively, the ~~Corporation~~Association may give notice to the Members stating that such documents are available at the registered office of the ~~Corporation~~Association and any Member may request a copy free of charge at the registered office or by prepaid mail.

2.05 **Corporate Seal**

The ~~Corporation~~Association may have a corporate seal in the form approved from time to time by the Board. If a corporate seal is approved by the Board, the Board shall determine which officer of the ~~Corporation~~Association shall be the custodian of the corporate seal.

2.06 **Execution of Documents**

Deeds, transfers, assignments, contracts, obligations and other instruments in writing requiring execution by the ~~Corporation~~Association may be signed by any two (2) of its officers or Directors. In addition, the Board may from time to time direct the manner in which and the person or persons by whom a particular document or type of document shall be executed. Any person authorized to sign any document may affix the corporate seal (if any) to the document. Any signing officer may certify a copy of any instrument, resolution, By-law or other document of the ~~Corporation~~Association to be a true copy thereof.

2.07 **Governance Policies**

The Board may adopt, amend, or repeal by resolution such Governance Policies that are not inconsistent with the By-laws of the ~~Corporation~~Association relating to various governance matters including ~~without limitation~~ terms of reference of committees, duties of Directors and officers as well as procedural and other requirements relating to the By-laws as the Board may deem appropriate from time to time. Any Governance Policies adopted by the Board will continue to have force and effect until amended, repealed, or replaced by a subsequent resolution of the Board.

2.08 **Affiliates**

The Board may approve categories of affiliates, including Student Affiliates, International Student Affiliates, International Affiliates, and Special Affiliates as further described in the Governance Policies. Affiliates shall not be Members of the ~~Corporation~~Association and shall have no voting privileges in the ~~Corporation~~Association. Persons qualified for membership in the ~~Corporation~~Association are not eligible for affiliate status. Affiliates shall enjoy the privileges set out in the Governance Policies. Affiliates shall comply with the Articles, By-laws and Governance Policies of the ~~Corporation~~Association.

SECTION 3 - MEMBERSHIP

3.01 **Classes and Conditions of Membership**

Pursuant to the Articles, there shall be one (1) class of Members in the ~~Corporation~~Association. Membership in the ~~Corporation~~Association shall be available to those who have applied for and been admitted into membership in the ~~Corporation~~Association in accordance with the Governance Policies. Applicants for membership must possess a Masters and/or Doctoral degree in psychology, conferred by a graduate school of recognized standing. Students of psychology, whether or not they have a qualifying degree, ~~shall~~ may apply to be Student Affiliates.

3.02 **Membership Transferability**

A membership may only be transferred to the ~~Corporation~~Association.

3.03 **Rights of Members**

A Member ~~of the CorporationAssociation~~ shall have the right to receive notice of, attend, speak and participate at all meetings of Members and the right to one (1) vote at all meetings of Members.

3.04 **Termination of Membership**

A membership in the ~~Corporation~~Association is terminated when:

(a) the Member dies;

(b) the Member fails to maintain the qualifications for membership set out in section 3.01;

(c) the Member resigns;

(d) the Member's term of membership expires, if applicable;

(e) the Member is removed by the Board in accordance with section 3.06;

Subject to the Articles, upon any termination of membership, the rights of the Member, including any rights in the property of the ~~Corporation~~Association, automatically cease to exist. Where a person is no longer a Member, then such person ~~[~~shall be deemed to have also automatically resigned as a Director~~] [NTD: This is probably not enforceable. There are ways to make this more enforceable. TBD.]~~, an officer and/or a committee member, as applicable, provided that the Board may in its discretion subsequently re-appoint such individual as a committee member if the Board deems it appropriate in the circumstances.

3.05 **Membership and Affiliate Dues**

The Directors may determine the amount and the manner in which membership and affiliate dues are to be paid. Members and affiliates shall be notified in writing of the dues at any time payable

by them and, if any are not paid within one (1) calendar month of the membership or affiliate renewal date, as the case may be, the Members and affiliates in default shall thereupon cease to be Members or affiliates of the ~~Corporation~~Association.

3.06 **Discipline of Members and Affiliates**

The Board may suspend or remove any Member or affiliate from the ~~Corporation~~Association for any one or more of the following grounds:

(a) violating any provision of the Articles, By-laws, ~~or~~ Governance Policies, or other policies and regulations of the ~~Corporation~~Association;

(b) carrying out any conduct which may be detrimental to the ~~Corporation~~Association as determined by the Board in its sole discretion;

(c) for any other reason that the Board in its sole and absolute discretion considers to be reasonable, having regard to the purposes of the ~~Corporation~~Association.

Suspension, expulsion and reinstatement of Members and affiliates shall take place in accordance with the provisions of the Governance Policies.

SECTION 4 - MEETINGS OF MEMBERS

4.01 **Notice of Meeting of Members**

In accordance with and subject to the Act, notice of the time and place of a meeting of Members shall be given to each Member entitled to vote at the meeting by the following means:

(a) by mail, courier or personal delivery to each Member entitled to vote at the meeting, during a period of 21 to 60 days before the day on which the meeting is to be held; or

(b) by telephonic, electronic or other communication facility to each Member entitled to vote at the meeting, during a period of 21 to 35 days before the day on which the meeting is to be held.

Notice of a meeting of Members shall also be given to each Director and to the public accountant of the ~~Corporation~~Association during a period of 21 to 60 days before the day on which the meeting it to be held. Notice of any meeting of Members at which special business (as defined in section 4.05) is to be transacted shall state the nature of that business in sufficient detail to permit the Member to form a reasoned judgment on the business and provide the text of any Special Resolution or By-law to be submitted to the meeting. The Directors may fix a record date for determination of Members entitled to receive notice of any meeting of Members in accordance with the requirements of section 161 of the Act. Subject to the Act, ~~a~~ notice of a Members' meeting ~~provided by the CorporationAssociation~~ shall include any Proposal submitted to the ~~Corporation~~Association under section 4.14.

4.02 **Annual Meetings**

An annual meeting of Members shall be held at such time in each year, as the Board may from time to time determine, provided that the annual meeting must be held not later than 15 months after holding the preceding annual meeting and no later than 6 months after the end of the ~~Corporation~~Association's preceding financial year. The annual meeting shall be held for the purpose of considering the financial statements and reports of the ~~Corporation~~Association

required by the Act to be presented at the meeting, electing Directors, appointing the public accountant and transacting such other business as may properly be brought before the meeting or is required under the Act. The Governance Policies may set out any other requirements, including but not limited to rules of procedure, relating to the holding of meetings of Members.

4.03 **Special Meetings**

The Board may at any time call a special meeting of Members for the transaction of any business which may properly be brought before the Members. In accordance with and subject to the Act, on written requisition by Members carrying not less than 5% of the votes that may be cast at a meeting of Members sought to be held, the Board shall call a special meeting of Members, unless the exceptions in the section 167 of the Act are met. If the Directors do not call a meeting within twenty-one (21) days of receiving the requisition, any Member who signed the requisition may call the meeting.

4.04 **Place of Meetings**

In accordance with and subject to the Act, meetings of Members may be held at any place within Canada as the Board may determine.

4.05 **Special Business**

All business transacted at a special meeting of Members and all business transacted at an annual meeting of Members, except consideration of the financial statements, public accountant's report, election of Directors and re-appointment of the incumbent public accountant, is "**special business**".

4.06 **Persons Entitled to be Present**

The only persons entitled to be present at a meeting of Members shall be those entitled to vote at the meeting, the Directors, the officers, and the public accountant of the ~~Corporation~~Association and such other persons who are entitled or required under any provision of the Act, Articles or By-laws to be present at the meeting. Any other person may be admitted only on the invitation of the chair of the meeting or by Ordinary Resolution of the Members.

4.07 **Chair of the Meeting**

The President shall act as chair of any meeting of Members. In the event that the President is absent, unable or unwilling to act, the incoming President or Immediate Past-President will chair the meeting, but if the incoming President or Immediate Past-President is absent, unable, or unwilling to act, the Members who are present and entitled to vote at the meeting shall choose one of their number to chair the meeting.

4.08 **Quorum**

Subject to the Act, a quorum at any meeting of the Members shall be at least twenty-five (25) Members present at the meeting. If a quorum is present at the opening of a meeting of Members, the Members present may proceed with the business of the meeting even if a quorum is not present throughout the meeting. For the purpose of determining quorum, a Member may be present in person or by telephonic and/or other electronic means. For greater certainty, Members who have cast their votes by electronic ballot (if applicable) shall also be counted as present for the purpose of determining quorum.

4.09 **Votes to Govern**

At any meeting of Members every question shall, unless otherwise provided by the Articles, By-laws or the Act, be determined by Ordinary Resolution. In case of an equality of votes either on a show of hands or on a ballot or on the results of electronic voting, the chair of the meeting shall not have a second or casting vote.

4.10 **Participation by Electronic Means**

If the ~~Corporation~~Association chooses to make available a telephonic, electronic or other communication facility that permits all participants to communicate adequately with each other during a meeting of Members, any person entitled to attend such meeting may participate in the meeting by means of such telephonic, electronic or other communication facility in the manner provided by the Act and the Regulations. A person participating in a meeting by such means is deemed to be present at the meeting. Notwithstanding any other provision of this By-law, any person participating in a meeting of Members pursuant to this section who is entitled to vote at that meeting may vote, in accordance with the Act and the Regulations, by means of any telephonic, electronic or other communication facility that the ~~Corporation~~Association has made available for that purpose.

4.11 **Meeting Held Entirely by Electronic Means**

Notwithstanding section 4.10, if the Directors or Members of the ~~Corporation~~Association call a meeting of Members, those Directors or Members, as the case may be, may determine that the meeting be held, in accordance with the Act and the Regulations, entirely by means of a telephonic, electronic or other communication facility that permits all participants to communicate adequately with each other during the meeting.

4.12 **Voting by Electronic Means**

Notwithstanding any other provision of this By-law, voting carried out by means of a telephonic, electronic or other communication facility referred to in section 4.10 and section 4.11 is permitted only if that facility enables the votes to be gathered in a manner that permits their subsequent verification; and permits the tallied votes to be presented to the ~~Corporation~~Association without it being possible for the ~~Corporation~~Association to identify how each Member voted.

4.13 **Electronic Ballots**

If the ~~Corporation~~Association makes available an electronic ballot system, Members who cannot attend a meeting of the Members may vote in advance of the meeting by electronic ballot in accordance with the instructions provided by the ~~Corporation~~Association, provided that any such system (a) enables the votes to be gathered in a manner that permits their subsequent verification; and (b) permits the tallied votes to be presented to the ~~Corporation~~Association without it being possible for the ~~Corporation~~Association to identify how each Member voted. An electronic ballot is valid only at the meeting in respect of which it is given or any adjournment thereof. Once tallied, the votes cast by electronic ballot will be added to the votes cast at the meeting to determine the outcome of the vote. No Member shall be entitled to vote in person or by electronic means at a meeting of Members if such Member has cast their vote in advance of the meeting by electronic ballot.

4.14 **Proposals at Annual Meetings**

Subject to compliance with section 163 of the Act, a Member entitled to vote at an annual meeting may submit to the ~~Corporation~~Association notice of any matter that the Member proposes to raise at the annual meeting (a "**~~proposal~~Proposal**"). Any such ~~proposal~~ Proposal may include nominations for the election of Directors if the ~~proposal~~ Proposal is signed by not less than five per cent (5%) of the Members entitled to vote at the meeting. Subject to the Act, the ~~Corporation~~Association shall include the ~~proposal~~ Proposal in the notice of meeting and if so requested by the Member, shall also include a statement by the Member in support of the ~~proposal~~ Proposal and the name and address of the Member. The Member who submitted the ~~proposal~~ Proposal shall pay the cost of including the ~~proposal~~ Proposal and any statement in the notice of meeting at which the ~~proposal~~ Proposal is to be presented unless otherwise provided by Ordinary Resolution of the Members present at the meeting.

SECTION 5 - DIRECTORS

5.01 Number of Directors

The Board shall consist of a number of Directors between the minimum and maximum number of Directors specified in the Articles. The precise number of Directors on the Board shall be determined from time to time by the Members by Ordinary Resolution, of, if the Ordinary Resolution empowers the Directors to determine the number of Directors, by resolution of the Board.

5.02 Qualifications

Each Director shall be an individual who is not less than 18 years of age. No person who has been found to be incapable (as the term "incapable" is defined in the Act) or who has the status of a bankrupt shall be a Director. Only Members shall be eligible to be elected and hold office as Directors of the ~~Corporation~~Association, except for the position representing Students referred to in section 5.03(e)~~, and any retiring Director who remains a Member of the CorporationAssociation may be eligible for re-election~~. No person shall be eligible to hold more than one position on the Board.

5.03 Composition of Board

Directors shall, as far as practicable, be representative of the geographical regions of Canada, and of gender, sub-discipline interest and language groups. The Board shall be comprised as far as possible of the following Directors, all of whom shall be elected by the Members except for those referred to in clause (a):

(a) The Directors appointed by the Board under section 5.~~07~~ 06 (if any);

(b) Three (3) Directors, of whom (i) one identifies as a scientist, (ii) one identifies as a practitioner, and (iii) one identifies as an educator;

(c) Five (5) Directors-at-large (including the President and Immediate Past-President);

(d) One (1) Director who is nominated by and from among the Council of Sections; and

(e) One (1) Director who is nominated by and from among the Section on Students.

For greater certainty, if a Director described under section 5.03(b) or section 5.03(d) is appointed as the President or Immediate Past-President, they will then be deemed to be a Director-at-large under section 5.03(c).

5.04 **<u>Election of Directors and Term</u>**

(a) Subject to the Articles and By-laws, Directors shall be elected by the Members by Ordinary Resolution at an annual meeting of Members at which an election of Directors is required.

(b) The terms of office of Directors shall be established by Ordinary Resolution of the Members provided that no Director shall hold office for longer than a four (4) year term.

(c) If Directors are not elected at a meeting of Members, the incumbent Directors shall continue in office until their successors are elected.

(d) Directors shall be eligible for re-election, except that no elected Director shall serve for more than two (2) consecutive terms, unless authorized by the Board in exceptional circumstances.

(e) The Board shall establish a Nominating Committee, the details of which shall be set forth herein and in the Governance Policies. The Nominating Committee will present a report to the Members for the election of Directors and such report will be prepared in accordance with the requirements of this By-law and the Governance Policies.

5.05 **<u>Nomination of Directors</u>**

(a) General - Subject to the Act, the Articles, and the By-laws, only persons who are nominated in accordance with the following procedures shall be eligible for election as Directors. Nominations of persons for election to the Board may be made by any Nominating Member in advance of any annual meeting of Members as set forth herein. For purposes of this section 5.05, a nominating Member ("**Nominating Member**") means a Member:

(i) who, at the close of business on the date of the giving of the notice provided for below and on the record date for notice of such meeting, is entered in the ~~Corporation~~<u>Association</u>'s membership records being entitled to vote at such meeting; and

(ii) who complies with the notice procedures set forth below.

(b) Timely Notice - In addition to any other applicable requirements, for a nomination to be valid, the Nominating Member must have submitted their nomination in proper written form to the ~~Secretary of the Corporation~~<u>Association</u>, in accordance with the instructions provided by the ~~Corporation~~<u>Association</u>, not less than 30 nor more than 65 days prior to the date of the annual meeting of Members.

(c) Proper Form - To be in proper written form, a Nominating Member's notice to the ~~Secretary~~ <u>Association</u> must set forth:

(i) as to each person whom the Nominating Member proposes to nominate for election as a Director: (A) the name and address of the person; (B) the principal occupation or employment of the person; and (C) any other information confirming that the person meets all of the qualification requirements of Directors set out in the By-laws of the ~~Corporation~~<u>Association</u> and such other applicable Governance Policies; and

(ii) as to the Nominating Member giving the notice, (A) the name and the residential address of the person; (B) the principal occupation or employment of the person;; and (C) confirmation that the person has the right to vote at the meeting of Members where the election is to be held;

provided that the ~~Corporation~~Association may also require any proposed nominee to furnish such other information, including a written consent to act, as may reasonably be required by the ~~Corporation~~Association to determine the eligibility of such proposed nominee to serve as a Director of the ~~Corporation~~Association.

(d) Validity - The Nominating Committee shall have the power and duty to determine whether a nomination was made in accordance with the requirements of this Section 5.05 and, if any proposed nomination is not in compliance, to declare that such nomination shall be disregarded.

(e) Delivery of Notice - Notwithstanding any other provision of this By-law, notice given to the ~~Secretary~~ Association ~~of the Corporation~~Association pursuant to this Section 5.05 may only be given by personal delivery, facsimile transmission or by email (at such email address as stipulated from time to time by the ~~Secretary~~ Association ~~of the Corporation~~Association for purposes of this notice), and shall be deemed to have been given and made only at the time it is served by personal delivery, email (at the aforesaid address) or sent by facsimile transmission (provided that receipt of confirmation of such transmission has been received) to the ~~Secretary at the address of the~~ registered office of the ~~Corporation~~Association; provided that if such delivery or electronic communication is made on a day which is a not a business day or later than 5:00 p.m. (EST) on a day which is a business day, then such delivery or electronic communication shall be deemed to have been made on the subsequent day that is a business day.

Notwithstanding the foregoing, the Board may, in its sole discretion, waive any requirement in this Section.

5.06 **Appointment of Directors and Term**

Subject to the By-laws, following the conclusion of the annual general meeting of Members each year, the Board may appoint one or more additional Directors; however the total number of Directors so appointed shall not exceed one-third (1/3) of the number of Directors elected by the Members at the previous annual meeting of Members. The appointed Directors will hold office as Directors and officers for a term expiring not later than the close of the next annual meeting of Members. Appointed Directors shall be eligible for re-appointment, except that no Director shall serve for more than six (6) consecutive terms, unless authorized by the Board in exceptional circumstances.

5.07 **Ceasing to Hold Office**

A Director ceases to hold office when the Director dies, resigns, is removed from office by the Members in accordance with section 5.09, or no longer fulfils all of the qualifications to be a Director set out in section 5.02 as determined in the sole discretion of the Board.

5.08 **Resignation**

In accordance with and subject to the Act, a resignation of a Director becomes effective at the time a written resignation is sent to the ~~Corporation~~Association or at the time specified in the resignation, whichever is later.

5.09 **Removal**

In accordance with and subject to the Act, the Members may, by Ordinary Resolution, passed at a meeting of Members, remove any Director from office before the expiration of the Director's term.

5.10 **Filling Vacancies**

In accordance with and subject to the Act and the Articles, a quorum of the Board may fill a vacancy in the Board, except a vacancy resulting from an increase in the number or the minimum or maximum number of Directors, or from a failure of the Members to elect the number of Directors required to be elected at any meeting of Members. If there is not a quorum of the Board, or if the vacancy has arisen from a failure of the Members to elect the number of Directors required to be elected at any meeting of Members, the Board shall forthwith call a special meeting of Members to fill the vacancy. If the Board fails to call such meeting or if there are no Directors then in office, any Member may call the meeting. A Director appointed or elected to fill a vacancy holds office for the unexpired term of their predecessor.

SECTION 6 - MEETINGS OF DIRECTORS

6.01 **Calling of Meetings**

Meetings of the Board may be called by the President or any three (3) Directors at any time.

6.02 **Notice of Meeting**

Notice of the time and place for the holding of a meeting of the Board shall be given in the manner provided in section 9.01 of this By-law to every Director not less than 48 hours before the time when the meeting is to be held. Notice of a meeting shall not be necessary if all of the Directors are present, and none objects to the holding of the meeting, or if those absent have waived notice of or have otherwise signified their consent to the holding of such meeting. Notice of an adjourned meeting is not required if the time and place of the adjourned meeting is announced at the original meeting. No notice of meeting need specify the purpose or the business to be transacted at the meeting except if subsection 136(3) of the Act requires the purpose thereof or the business to be transacted to be specified in the notice.

6.03 **Regular Meetings**

The Board may appoint a day or days in any month or months for regular meetings of the Board at a place and hour to be named. A copy of any resolution of the Board fixing the place and time of such regular meetings of the Board shall be sent to each Director forthwith after being passed, but no other notice shall be required for any such regular meeting except if subsection 136(3) of the Act requires the purpose thereof or the business to be transacted to be specified in the notice.

6.04 **Participation at Meeting by Telephone or Electronic Means**

If all of the Directors consent, a Director may, in accordance with the Regulations, participate in a Board meeting, by means of a telephonic, electronic or other communications facility that permits all participants to communicate adequately with each other during the meeting. A Director participating in the meeting by such means shall be deemed for the purposes of the Act to have been present at that meeting. A consent pursuant to this section may be given before or after the

meeting to which it relates and may be given with respect to all meetings of the Board and committees of the Board.

6.05 **Votes to Govern**

At all meetings of the Board, every question shall be decided by a majority of the votes cast on the question. In case of an equality of votes, the chair of the meeting shall not have a second or casting vote.

6.06 **Quorum**

A quorum shall be a majority of the number of Directors determined in accordance with section 5.01. For the purpose of determining quorum, a Director may be present in person, or, if authorized under this By-law, by teleconference and/or by other electronic means.

6.07 **Resolutions in Writing**

A resolution in writing, signed by all the Directors entitled to vote on that resolution at a Board meeting, shall be as valid as if it had been passed at a Board meeting. A copy of every such resolution in writing shall be kept with the minutes of the proceedings of the Board or committee of Directors.

6.08 **Board Committees and Advisory Bodies**

Subject to the Act, the Board may from time to time appoint any committee or other advisory body, as it deems necessary or appropriate for such purposes, subject to the Act, with such powers as the Board shall see fit. Any committee member may be removed by the Board. Unless otherwise determined by the Board, a committee shall have the power to fix its quorum at not less than a majority of its members, to elect its chairperson and to otherwise regulate its procedure. Terms of reference of all Board committees~~, including the Finance and Audit Committee, Nominations Committee, Governance Committee, and Committee on Fellows and Awards~~ shall be set out in the Governance Policies.

6.09 **Partner Representatives**

(a) The Board will designate up to four (4) organizations as partner organizations who each shall be entitled to appoint an individual to be its partner representative to represent it at meetings of the Board.

(b) The partner representatives may be invited to participate in Board meetings or portions thereof. For greater certainty, the partner representatives are not Directors and shall not be entitled to vote and shall not be counted for quorum purposes at any meeting of the Board.

(c) An individual shall become a partner representative effective upon the date of the appointment, and shall cease to be a partner representative upon the date that the appointment of their successor is effective.

6.10 **Disclosure of Interest**

Without limiting the requirements of the Act or any Governance Policies, every Director and officer shall disclose to the ~~Corporation~~Association the nature and extent of any interest that the Director

or officer has in a material contract or material transaction, whether made or proposed, with the ~~Corporation~~Association, in accordance with the manner and timing provided in the Act.

SECTION 7 - OFFICERS

7.01 **Appointment**

The Board may designate the offices of the ~~Corporation~~Association, appoint officers, specify their duties and, subject to the Act, delegate to such officers the power to manage the affairs of the ~~Corporation~~Association. A Director may be appointed to any office of the ~~Corporation~~Association, except that the Director described in ~~[~~section 5.03(a) or 5.03(e)~~]~~ is not eligible to be appointed the President or Immediate Past-President. An officer may, but need not be, a Director unless these ~~by~~By-laws otherwise provide. The term of office of officers shall be one (1) year unless otherwise determined by the Board by resolution.

7.02 **Description of Offices**

Unless otherwise specified by the Board (which may, subject to the Act, modify, restrict or supplement such duties and powers), the offices of the ~~Corporation~~Association, if designated and if officers are appointed thereto, shall have the following duties and powers associated therewith, as well as such other duties and powers as the Board may specify from time to time:

(a) **Honorary President** – An Honorary President may be appointed by the Board for a period of one year. The duties of the Honorary President shall be determined by the Board.

(b) **President** - The President shall be a Member and Director of the ~~Corporation~~Association. The President shall act as chair of meetings of Members and the Board and shall see that all orders and resolutions of the Board are carried into effect. The President shall also be a member ex-officio of all committees of the Board. The President shall have such other powers and shall perform such other duties as may from time to time be assigned to the President by resolution of the Board or as are set forth in the Governance Policies.

(c) **Immediate Past-President** – The Immediate Past-President shall be a Member and Director of the ~~Corporation~~Association and shall be appointed at the end of the term of the incumbent President. The duties of the Immediate Past-President shall be determined by the Board.

(d) **Chief Executive Officer** – The Chief Executive Officer shall be appointed by the Board to hold office until the termination of their contract and upon completion of all duties connected therewith.

The Chief Executive Officer shall have full authority subject to the authority of the Board, to manage and direct the affairs of the ~~Corporation~~Association. The Chief Executive Officer shall conform to all lawful orders given to them by the Board. The Chief Executive Officer shall give to the Directors, to the officers or any one of them, from time to time, all information they may require regarding the affairs of the ~~Corporation~~Association. The Chief Executive Officer shall be responsible for the recording of all votes and minutes of all proceedings in the books to be kept for that purpose.

The Chief Executive Officer shall be responsible for keeping a register of Members and shall have custody of the corporate seal of the ~~Corporation~~Association. The Chief Executive Officer shall be responsible for preparing an annual budget for all accounts, for submission to the Board for its consideration.

The Chief Executive Officer shall have custody of the corporate funds and securities and shall be responsible for keeping full and accurate accounts of receipts and disbursements in books belonging to the ~~Corporation~~Association, and shall be responsible for the deposit of all monies and other valuable effects in the name and to the credit of the ~~Corporation~~Association and in such depository as may be designated by the Board from time to time. The Chief Executive Officer shall be responsible for the disbursement of the funds of the ~~Corporation~~Association as may be ordered by the Board, and for taking proper vouchers for such disbursements, and shall render to the Board whenever they may require it, an account of all transactions and of the financial position of the ~~Corporation~~Association.

The powers and duties of all other officers of the ~~Corporation~~Association shall be such as the terms of their engagement call for or the Board or President requires of them. The Board may from time to time and subject to the Act, vary, add to or limit the powers and duties of any officer.

7.03 **Vacancy in Office**

In the absence of a written agreement to the contrary, the Board may remove, whether for cause or without cause, any officer of the ~~Corporation~~Association. Unless so removed, an officer shall hold office until the earlier of:

(a) the officer's successor being appointed,

(b) the officer's resignation,

(c) such officer ceasing to be a Director (if a necessary qualification of appointment) or

(d) such officer's death.

If the office of any officer of the ~~Corporation~~Association shall be or become vacant, the Board may, by resolution, appoint a person to fill such vacancy.

SECTION 8 - SECTIONS

8.01 **Purpose**

Sections are the primary agents through which the particular and special needs of Members are met and interests are served. Those matters pertaining to the discipline of psychology as a whole are the responsibility of the Board~~ of the Corporation~~~~Association~~. The Board may establish Governance Policies including rules and regulations concerning the formation, operation, conduct, and dissolution of Sections and may delegate the development and implementation of section operating regulations to the Chief Executive Officer.

8.02 **Formation**

Approval to establish a Section within the ~~Corporation~~Association may be granted by the Board when a group of at least 25 ~~Fellows and ~~Members of the ~~Corporation~~Association submit a petition to the Board which includes a statement of purpose and goals of the proposed Section.

8.03 **Dissolution and Restructuring**

A Section of the ~~Corporation~~Association may be dissolved by the Board (a) if the Section breaches any Governance Policies; (b) when the Section votes to request dissolution or when the number

of ~~Fellows and~~ Members of the Section falls below 25 ~~Fellows and~~ Members.<u> Without limiting the generality of the foregoing or any Governance Policies, the Board may, instead of dissolving a Section, remove and replace the members of the Section executive in the event that the Section breaches any Governance Policies.</u> In the event that the number of ~~Fellows and~~ Members of the Section falls below 25 ~~Fellows and~~ Members, the Section may submit a petition to the Board to stay dissolution for one year to enable the Section to re-establish the necessary number of ~~Fellows and~~ Members. If the Section is unable to achieve the required level within one year the Section shall be dissolved.

8.04 **<u>Organization</u>**

Within twelve months of obtaining approval to establish a Section (or within twelve months of the Board approving any amendments to the Model Terms of Reference described herein), the Section shall pass terms of reference in conformity with such Model Terms of Reference as may from time to time be approved by the Board of the ~~Corporation~~<u>Association</u> and the Section terms of reference will take effect upon approval by the Board or its designate.

8.05 **<u>Regulation of Section Affairs</u>**

A Section may engage in activities <u>of relevance to its members, </u>and<u> may</u> adopt rules, regulations and policies for the management of its affairs ~~which~~ <u>that </u>are not inconsistent with the Articles, By-laws<u>,</u> ~~or~~ Governance Policies<u> or section operating regulations</u> of the ~~Corporation~~<u>Association</u>. For greater certainty, nothing herein shall be construed as giving to a Section, the power and authority to:

(a) contract a loan, open a line of credit or issue a guarantee without the prior approval of the Board;

(b) make any expense or disbursement, or incur any contractual liability which is in excess of the amounts approved by the Board from time to time<u> or as otherwise set out in the Governance Policies or section operating regulations</u>;<u> or</u>

(c) make<u> any</u> public statements,<u> issue position papers or policy statements, or otherwise</u> represent or act on behalf of the Section or the ~~Corporation~~<u>Association </u> ~~in any manner or thing, except~~ with<u>out</u> the express written consent of the ~~Board of the Corporation~~<u>Association</u>.

8.06 **<u>Dues and Fees Invoicing</u>**

Sections may establish annual section dues which will be collected by the central office. Sections shall have the authority to solicit donations from their Members for specific identified purposes.

8.07 **<u>Reports</u>**

At least ~~four~~ <u>eight </u>weeks before the annual meeting of the ~~Corporation~~<u>Association</u>, the secretary of each Section shall submit an annual ~~report~~ <u>update </u>to the Board of the ~~Corporation~~<u>Association</u> that contains the information prescribed by the ~~Board~~<u>Association</u>. ~~The annual report shall include a budget for the ensuing year which shall be subject to approval by the Board.~~

8.08 **Council of Sections**

The chair of each Section shall form the Council of Sections of the ~~Corporation~~Association. The Council of Sections will provide guidance to the Board or the Chief Executive Officer from time to time and perform such other duties as may be specified in the Governance Policies.

~~8.09 **Powers**~~

~~With the prior written approval of the Board or its designate in each instance, a section shall have the power to initiate and undertake activities of relevance to its Members, to draft position papers on topics of relevance to the Section, to initiate policy statements in areas of expertise and to organize meetings. A Section may make specific representations to external organizations or agencies only if it has received the approval of the Board to do so, or it may recommend that the~~ ~~Corporation~~~~Association~~ ~~make such representations.~~

~~8.10~~8.09 **Access**

A Section shall have access to the Board through the Director described at section 5.03(d) for consideration of issues of concern to the Section involving matters relevant to the relationship of the Section to the ~~Corporation~~Association as a whole or component thereof, or concerning agencies or activities external to the ~~Corporation~~Association.

SECTION 9 - NOTICES

9.01 **Method of Giving Notices**

Subject to section 4.01 and 6.02, any notice to be given (which term includes sent, delivered or served) pursuant to the Act, the Articles, the By-laws or otherwise to a Member, Director, officer, member of a committee of the Board, or the public accountant shall be sufficiently given:

(a) if delivered personally to the person to whom it is to be given or if delivered to such person's address as shown in the records of the ~~Corporation~~Association or in the case of notice to a Director to the latest address as shown in the last notice that was filed by the ~~Corporation~~Association in accordance with the Act and received by Corporations Canada;

(b) if mailed to such person at such person's recorded address by prepaid ordinary or air mail;

(c) if sent to such person by telephonic, electronic or other communication facility at such person's recorded address for that purpose; or

(d) if provided in the form of an electronic document in accordance with the Act.

A notice so delivered shall be deemed to have been given when it is delivered personally or to the recorded address as aforesaid; a notice so mailed shall be deemed to have been given when deposited in a post office or public letter box; and a notice so sent by any means of transmitted or recorded communication shall be deemed to have been given when dispatched or delivered to the appropriate communication company or agency or its representative for dispatch.

The ~~Secretary~~ Association may change or cause to be changed the recorded address of any Member, Director, officer, public accountant, or member of a committee of the Board in accordance with any information believed by the ~~Secretary~~ Association to be reliable. ~~The~~

~~declaration by the Secretary that notice has been given pursuant to this By-law shall be sufficient and conclusive evidence of the giving of such notice.~~ The signature of any Director or officer of the ~~Corporation~~Association to any notice or other document to be given by the ~~Corporation~~Association may be written, stamped, type-written or printed or partly written, stamped, type-written or printed.

9.02 **Computation of Time**

If a given number of days' notice or notice extending over a period is required to be given under the By-laws, the day of service, posting or other delivery of the notice shall not, unless it is otherwise provided, be counted in such number of days or other period.

9.03 **Undelivered Notices**

If any notice given to a Member is returned on two consecutive occasions because such Member cannot be found, the ~~Corporation~~Association shall not be required to give any further notices to such Member until such Member informs the ~~Corporation~~Association in writing of their new address.

9.04 **Omissions and Errors**

The accidental omission to give any notice to any Member, Director, officer, member of a committee of the Board or public accountant, or the non-receipt of any notice by any such person or any error in any notice not affecting its substance shall not invalidate any action taken at any meeting to which the notice pertained or otherwise founded on such notice.

9.05 **Waiver of Notice**

Any Member, Director, officer, member of a committee of the Board or public accountant may waive or abridge the time for any notice required to be given to such person, and such waiver or abridgement, whether given before or after the meeting or other event of which notice is required to be given shall cure any default in the giving or in the time of such notice, as the case may be. Any such waiver or abridgement shall be in writing except a waiver of notice of a meeting of Members or of the Board or of a committee of the Board, which may be given in any manner.

SECTION 10 - BY-LAWS

10.01 **Amendment of Articles**

The Articles of the ~~Corporation~~Association may only be amended if the amendment is sanctioned by a Special Resolution of the Members. Any amendment to the Articles is effective on the date shown in the certificate of amendment.

10.02 **Effective Date of Board Initiated By-law, Amendment or Repeal**

Subject to the Act and section 10.03 of this By-law, the Board may, by resolution, make, amend or repeal any By-laws that regulate the activities or affairs of the ~~Corporation~~Association. Any such By-law, amendment or repeal shall be effective from the date of the resolution of Directors until the next meeting of Members where it may be confirmed, rejected or amended by the Members by Ordinary Resolution. If the By-law, amendment or repeal is confirmed or confirmed as amended by the Members it remains effective in the form in which it was confirmed. The By-law, amendment or repeal ceases to have effect if it is not submitted to the Members at the next meeting of Members or if it is rejected by the Members at the meeting.

10.03 **Effective Date of By-law, Amendment or Repeal under Subsection 197(1)**

A By-law made, amended or repealed under subsection 197(1) of the Act is effective from the date of the Special Resolution of Members approving such By-law, amendment or repeal.

10.04 **Repeal of Previous By-laws**

All previous by-laws of the ~~Corporation~~Association related to the subject matter of this By-law are repealed as of the effective date of this By-law. Such repeal shall not affect the validity of any act done or right or privilege, obligation or liability acquired or incurred under such By-law prior to its repeal. All Directors, officers, committee members, and persons acting under any by-law so repealed shall continue to act as if elected or appointed under the provisions of this By-law, and all resolutions of the Directors or Members with continuing effect passed under any repealed By-law shall continue to be good and valid except to the extent inconsistent with this By-law and until amended or repealed.

Adopted by the Special Meeting of the Canadian Psychological Association, [•], 2025
