# Supplementary material for: CPA governance restructuring and section autonomy: a case study in organizational resistance to structural diversity
Source: Int J Ment Health Syst. 2026 Jul 30;20:26. doi: 10.1186/s13033-026-00722-z (PMC13459331; doi:10.1186/s13033-026-00722-z)
Supplement: Supplementary file 4 [file 13033_2026_722_MOESM4_ESM.pdf]

CANADIAN PSYCHOLOGICAL ASSOCIATION
SOCIÉTÉ CANADIENNE DE PSYCHOLOGIE

Amended: October 2018[•], 2025

BY-LAW NO. 1

A byBy-law relating generally to the conduct of the affairs of

CANADIAN PSYCHOLOGICAL ASSOCIATION
SOCIÉTÉ CANADIENNE DE PSYCHOLOGIE

(the "Corporation**Association**")

TABLE OF CONTENTS

Section 1 - Interpretation
Section 2 - Financial and Other Matters
Section 3 - Membership
Section 4 - Meetings of Members
Section 5 - Directors
Section 6 - Meetings of Directors
Section 7 - Officers
Section 8 - Sections
Section 9 - Notices
Section 10 - By-laws

WHEREAS the Corporation was granted Letters Patent by the federal Government of Canada under the *Canada Corporations Act* on the 16th day of August, 1962;

AND WHEREAS the Corporation has applied for a certificate of continuance to be continued under the *Canada Not for Profit Corporations Act* S.C. 2009, c.23;

NOW THEREFORE BE IT ENACTED as a general operating By-law of the Corporation to take effect in accordance with section 11.01Association as follows:

SECTION 1 - INTERPRETATION

1.01 **Definitions**

In this byBy-law and all other by-laws of the Corporation, unless the context otherwise requires:

(a) "**Act**" means the *Canada Not-For_____ Profitfor-profit Corporations Act* S.C. 2009, c.23 including the Regulations made pursuant to the Act, and any statute or regulations that may be substituted, as amended from time to time;

(b) "articles**Articles**" means the original or restated articles of incorporation or articles of amendment, amalgamation, continuance, reorganization, arrangement or revival of the CorporationAssociation;

(c) "boardBoard" means the boardBoard of directorsDirectors of the CorporationAssociation and "directorDirector" means a member of the boardBoard;

( ) "byBy-lawlaws" means this byBy-law No. 1 and any other by-lawlaws of the CorporationAssociation as amended and which are, from time to time, in force and effect;

(d) "**Fellows**" means membersMembers who have made a distinguished contribution to the advancement of the science or profession of psychology or who have given exceptional service to their national or provincial associations, have met the requirements set forth in Operating RegulationsGovernance Policies, who have been recommended as Fellows by the Committee on Fellows and Awards and who have been approved as such by the boardBoard;

( ) "meeting of members" includes an annual meeting of members or a special meeting of members;

(f) (g) "Operating Regulations**Governance Policies**" means the operating regulationsgovernance policies approved by the boardBoard in accordance with section 2.07 of this byBy-law;

(a) "ordinary resolution**Member**" means any person who has been admitted to membership in the Association in accordance with the By-laws;

(a) "**Ordinary Resolution**" means a resolution passed by a majority of not less than 50% plus 1 of the votes cast on that resolution;

(b) "proposal" means a proposal submitted by a member of the Corporation that meets the requirements of section 163 of the Act**Proposal**" has the meaning given in section 4.14 of this By-law;

(e) "**Regulations**" means the regulations made under the Act, as amended, restated or in effect from time to time;

(f) "**Section**" means a section of the Corporation has the meaning set outAssociation, as described in section 8 of this byBy-law; and

(c) "special resolution**Special Resolution**" means a resolution passed by a majority of not less than two-thirds (2/3) of the votes cast on that resolution.

1.02 **Interpretation**

The following rules of interpretation will apply to this byBy-law, (unless the context otherwise requires):

(a) words importing the singular number only will include the plural and *vice versa*;

(b) the word "person" will include an individual, sole proprietorship, partnership, unincorporated association, body corporate, and a natural person;

(c) words referring to a gender include the feminine, masculine and neuterall genders; and

(d) the by lawsif any of the Corporation will be strictly interpreted at all times in accordance with and subject to the purposesprovisions contained in the By-laws are inconsistent with

those contained in the articles of Articles or the Corporation Act, the provisions contained in the Articles or the Act, as the case may be, shall prevail.

SECTION 2 - FINANCIAL AND OTHER MATTERS

2.01 **Financial Year**

Unless otherwise changed by resolution of the boardBoard, the financial year end of the CorporationAssociation shall be the 31st day of December in each year.

2.02 **Banking Arrangements**

The banking business of the CorporationAssociation shall be transacted at such bank, trust company or other firm or corporation carrying on a banking business in Canada or elsewhere as the board of directorsBoard may designate, appoint or authorize from time to time by resolution. The banking business or any part of it shall be transacted by an officer or officers of the CorporationAssociation and/or other persons as the board of directorsBoard may by resolution from time to time designate, direct or authorize.

2.03 **Public Accountant**

2.04 The Corporation shall be subject to the requirements relating to appointment of a public accountant and level of financial review required by the Act. The members may resolve not to appoint a public accountant if the Corporation meets the requirements for a "designated corporation" under the Act (by having $50,000 or less in gross annual revenues for its last completed financial year) and upon unanimous approval of the members.

At each annual meeting, the Members shall appoint a public accountant to hold office until the close of the next annual meeting and, if an appointment is not so made, the public accountant in office will continue in office until a successor is appointed. The Members may, at any special meeting, remove the public accountant before the expiration of such public accountant's term of office, and shall, by a majority of the votes cast at that meeting, appoint another public accountant in such public accountant's place for the remainder of such public accountant's term. If the Members fail to appoint a successor public accountant, the Directors shall immediately fill any vacancy in the office of public accountant. The remuneration of the public accountant shall be fixed by the Board.

2.04 2.05 **Annual Financial Statements**

The CorporationAssociation shall send copies of the annual financial statements and other documents referred to in subsection 172(1) of the Act to the membersMembers between 21 to 60 days before the day on which an annual meeting of membersMembers is held or before the day on which a written resolution in lieu of an annual meeting is signed, unless a memberMember declines to receive them. Alternatively, the CorporationAssociation may give notice to the membersMembers stating that such documents are available at the registered office of the CorporationAssociation and any memberMember may request a copy free of charge at the registered office or by prepaid mail.

2.05 2.06 **Corporate Seal**

The CorporationAssociation may have a corporate seal in the form approved from time to time by the boardBoard. If a corporate seal is approved by the boardBoard, the boardBoard shall

determine which officer of the ~~Corporation~~Association shall be the custodian of the corporate seal.

2.06 ~~2.07~~ **Execution of Documents**

Deeds, transfers, assignments, contracts, obligations and other instruments in writing requiring execution by the ~~Corporation~~Association may be signed by any two (2) of its officers or ~~directors~~Directors. In addition, the ~~board~~Board may from time to time direct the manner in which and the person or persons by whom a particular document or type of document shall be executed. Any person authorized to sign any document may affix the corporate seal (if any) to the document. Any signing officer may certify a copy of any instrument, resolution, ~~by~~By-law or other document of the ~~Corporation~~Association to be a true copy thereof.

~~2.08 **Operating Regulations**~~

2.07 **Governance Policies**

The ~~board~~Board may adopt, amend, or repeal by resolution such ~~operating regulations~~Governance Policies that are not inconsistent with the ~~by~~By-laws of the ~~Corporation~~Association relating to ~~such~~various governance matters as including terms of reference of committees, duties of Directors and officers, ~~board code of conduct and conflict of interest~~ as well as procedural and other requirements relating to the ~~by~~By-laws as the ~~board~~Board may deem appropriate from time to time. Any ~~Operating Regulations~~Governance Policies adopted by the ~~board~~Board will continue to have force and effect until amended, repealed, or replaced by a subsequent resolution of the ~~board~~Board.

2.08 ~~2.09~~ **Affiliates**

The ~~board of directors of the Corporation~~Board may approve categories of affiliates, including Student Affiliates, International Student Affiliates, International Affiliates, and Special Affiliates ~~and Honorary Fellows~~ as further described in the ~~Corporation's Operating Regulations~~Governance Policies. Affiliates shall not be ~~members~~Members of the ~~Corporation~~Association and shall have no voting privileges in the ~~Corporation~~Association. Persons qualified for membership in the ~~Corporation~~Association are not eligible for affiliate status. Affiliates shall enjoy the privileges set out in the ~~Operating Regulations~~Governance Policies. Affiliates shall comply with the ~~articles~~Articles, ~~by~~By-laws and ~~Operating Regulations~~Governance Policies of the ~~Corporation~~Association.

SECTION 3 - MEMBERSHIP

3.01 **Classes and Conditions of Membership**

Pursuant to the Articles, there shall be one (1) class of ~~members~~Members in the ~~Corporation~~Association. Membership in the ~~Corporation~~Association shall be available to those who have applied for and been admitted into membership in the ~~Corporation~~Association in accordance with the ~~Corporation's Operating Regulations~~Governance Policies. Applicants for membership must possess a Masters and/or Doctoral degree in psychology conferred by a graduate school of recognized standing.

Students of psychology, whether or not they have a qualifying degree, ~~shall~~may apply to be Student Affiliates.

3.02 **Membership Transferability**

A membership may only be transferred to the ~~Corporation~~Association.

3.03 **Rights of Members**

A~~ member of the Corporation~~Member shall have the right to receive notice of, attend, speak and participate at all meetings of ~~members~~Members and the right to one (1) vote at all meetings of ~~members~~Members.

3.04 **Termination of Membership**

A membership in the ~~Corporation~~Association is terminated when:

(a) the ~~member~~Member dies;

(b) the ~~member~~Member fails to maintain the qualifications for membership set out in section 3.01;

(c) the ~~member~~Member resigns;

(d) the ~~member~~Member's term of membership expires, if applicable;

(e) the ~~member~~Member is removed by the ~~board~~Board in accordance with section 3.06;

Subject to the ~~articles~~Articles, upon any termination of membership, the rights of the ~~member~~Member, including any rights in the property of the ~~Corporation~~Association, automatically cease to exist. Where a person is no longer a ~~member~~Member, then such person shall be deemed to have also automatically resigned as a ~~director~~Director, an officer and/or a committee member, as applicable, provided that the ~~board~~Board may in its discretion subsequently re-appoint such individual as a committee member if the ~~board~~Board deems it appropriate in the circumstances.

3.05 **Membership and Affiliate Dues**

The ~~directors~~Directors may determine the amount and the manner in which membership and affiliate dues are to be paid. Members and affiliates shall be notified in writing of the dues at any time payable by them and, if any are not paid within one (1) calendar month of the membership or affiliate renewal date, as the case may be, the ~~members~~Members and affiliates in default shall thereupon cease to be ~~members~~Members or affiliates of the ~~Corporation~~Association.

3.06 **Discipline of Members and Affiliates**

The ~~board~~Board may suspend or remove any ~~member~~Member or affiliate from the ~~Corporation~~Association for any one or more of the following grounds:

(a) violating any provision of the ~~articles~~Articles, ~~by~~By-laws,~~ or Operating Regulations~~Governance Policies, or other policies and regulations of the ~~Corporation~~Association;

(b) carrying out any conduct which may be detrimental to the ~~Corporation~~Association as determined by the ~~board~~Board in its sole discretion;

(c) for any other reason that the ~~board~~Board in its sole and absolute discretion considers to be reasonable, having regard to the ~~objects~~purposes of the ~~Corporation~~Association.

Suspension, expulsion and reinstatement of ~~members~~Members and affiliates shall take place in accordance with the provisions~~ set forth in~~of the~~ Operating Regulations~~Governance Policies.

SECTION 4 - MEETINGS OF MEMBERS

4.01 **Notice of Meeting of Members**

In accordance with and subject to the Act, notice of the time and place of a meeting of ~~members~~Members shall be given to each ~~member~~Member entitled to vote at the meeting by the following means:

(a) by mail, courier or personal delivery to each ~~member~~Member entitled to vote at the meeting, during a period of 21 to 60 days before the day on which the meeting is to be held; or

(b) by telephonic, electronic or other communication facility to each ~~member~~Member entitled to vote at the meeting, during a period of 21 to 35 days before the day on which the meeting is to be held.

Notice of a meeting of ~~members~~Members shall also be given to each ~~director~~Director and to the public accountant of the ~~Corporation~~Association during a period of 21 to 60 days before the day on which the meeting it to be held. Notice of any meeting of ~~members~~Members at which special business (as defined in section 4.05) is to be transacted shall state the nature of that business in sufficient detail to permit the ~~member~~Member to form a reasoned judgment on the business and provide the text of any~~ special resolution~~Special Resolution or ~~by~~By-law to be submitted to the meeting. The ~~directors~~Directors may fix a record date for determination of ~~members~~Members entitled to receive notice of any meeting of ~~members~~Members in accordance with the requirements of section 161 of the Act. Subject to the Act, a notice of ~~members~~a Members' meeting~~ provided by the Corporation~~ shall include any ~~proposal~~Proposal submitted to the ~~Corporation~~Association under section 4.14.

4.02 **Annual Meetings**

An annual meeting of ~~members~~Members shall be held at such time in each year, as the ~~board~~Board may from time to time determine, provided that the annual meeting must be held not later than 15 months after holding the preceding annual meeting and no later than 6 months after the end of the ~~Corporation~~Association's preceding ~~fiscal~~financial year. The annual meeting shall be held for the purpose of considering the financial statements and reports of the ~~Corporation~~Association required by the Act to be presented at the meeting, electing ~~directors~~Directors, appointing the public accountant and transacting such other business as may properly be brought before the meeting or is required under the Act. The~~ Operating Regulations~~Governance Policies may set out any other requirements, including but not limited to rules of procedure, relating to the holding of~~ annual~~ meetings of ~~members~~Members.

4.03 **Special Meetings**

The ~~board~~Board may at any time call a special meeting of ~~members~~Members for the transaction of any business which may properly be brought before the ~~members~~Members. In accordance with and subject to the Act, on written requisition by ~~members~~Members carrying not less than 5% of the votes that may be cast at a meeting of ~~members~~Members sought to be held, the ~~board~~Board shall call a special~~ general~~ meeting of ~~members~~Members, unless the exceptions in the section

167 of the Act are met. If the ~~directors~~Directors do not call a meeting within twenty-one (21) days of receiving the requisition, any ~~member~~Member who signed the requisition may call the meeting.

4.04 **Place of Meetings**

In accordance with and subject to the Act, meetings of ~~members~~Members may be held at any place within Canada as the ~~board~~Board may determine~~ or outside Canada if all of the members entitled to vote at such meeting so agree~~.

4.05 **Special Business**

All business transacted at a special meeting of ~~members~~Members and all business transacted at an annual meeting of ~~members~~Members, except consideration of the financial statements, public accountant's report, election of ~~directors~~Directors and re-appointment of the incumbent public accountant, is "**special business**".

4.06 **Persons Entitled to be Present**

The only persons entitled to be present at a meeting of ~~members~~Members shall be those entitled to vote at the meeting, the ~~directors~~Directors, the officers, and the public accountant of the ~~Corporation~~Association and such other persons who are entitled or required under any provision of the Act, ~~articles~~Articles or ~~by~~By-laws~~ of the Corporation~~ to be present at the meeting. Any other person may be admitted only on the invitation of the chair of the meeting or by~~ ordinary resolution~~Ordinary Resolution of the ~~members~~Members.

4.07 **Chair of the Meeting**

The President shall act as chair of any meeting of ~~members~~Members. In the event that the President is absent,~~ the members~~unable or unwilling to act, the incoming President or Immediate Past-President will chair the meeting, but if the incoming President or Immediate Past-President is absent, unable, or unwilling to act, the Members who are present and entitled to vote at the meeting shall choose one of their number to chair the meeting.

4.08 **Quorum**

Subject to the Act, a quorum at any meeting of the ~~members~~Members shall be at least twenty-five (25) ~~members~~Members present at the meeting. If a quorum is present at the opening of a meeting of ~~members~~Members, the ~~members~~Members present may proceed with the business of the meeting even if a quorum is not present throughout the meeting. For the purpose of determining quorum, a ~~member~~Member may be present in person or by telephonic and/or other electronic means. For greater certainty, ~~members~~Members who have cast their votes by electronic ballot (if applicable) shall also be counted as present for the purpose of determining quorum.

4.09 **Votes to Govern**

At any meeting of ~~members~~Members every question shall, unless otherwise provided by the ~~articles or by~~Articles, By-laws or by the Act, be determined by~~ ordinary resolution~~Ordinary Resolution. In case of an equality of votes either on a show of hands or on a ballot or on the

results of electronic voting, the chair of the meeting ~~in addition to an original vote~~ shall not have a second or casting vote.

4.10 **Participation by Electronic Means**

If the ~~Corporation~~Association chooses to make available a telephonic, electronic or other communication facility that permits all participants to communicate adequately with each other during a meeting of ~~members~~Members, any person entitled to attend such meeting may participate in the meeting by means of such telephonic, electronic or other communication facility in the manner provided by the Act and the Regulations. A person participating in a meeting by such means is deemed to be present at the meeting. Notwithstanding any other provision of this ~~by~~By-law, any person participating in a meeting of ~~members~~Members pursuant to this section who is entitled to vote at that meeting may vote, in accordance with the Act and the Regulations, by means of any telephonic, electronic or other communication facility that the ~~Corporation~~Association has made available for that purpose.

4.11 **Meeting Held Entirely by Electronic Means**

Notwithstanding section 4.10, if the ~~directors~~Directors or ~~members~~Members of the ~~Corporation~~Association call a meeting of ~~members~~Members, those ~~directors~~Directors or ~~members~~Members, as the case may be, may determine that the meeting be held, in accordance with the Act and the Regulations, entirely by means of a telephonic, electronic or other communication facility that permits all participants to communicate adequately with each other during the meeting.

4.12 **Voting by Electronic Means**

Notwithstanding any other provision of this ~~by~~By-law, voting carried out by means of a telephonic, electronic or other communication facility referred to in section 4.10 and section 4.11 is permitted only if that facility enables the votes to be gathered in a manner that permits their subsequent verification; and permits the tallied votes to be presented to the ~~Corporation~~Association without it being possible for the ~~Corporation~~Association to identify how each ~~member~~Member voted.

4.13 **Electronic Ballots**

~~Without limiting section 5.05, if~~If the ~~Corporation~~Association makes available an electronic ballot system, ~~members~~Members who cannot attend a meeting of the ~~members~~Members may vote in advance of the meeting by electronic ballot in accordance with the instructions provided by the ~~Corporation~~Association, provided that any such system (a) enables the votes to be gathered in a manner that permits their subsequent verification; and (b) permits the tallied votes to be presented to the ~~Corporation~~Association without it being possible for the ~~Corporation~~Association to identify how each ~~member~~Member voted. An electronic ballot is valid only at the meeting in respect of which it is given or any adjournment thereof. Once tallied, the votes cast by electronic ballot will be added to the votes cast at the meeting to determine the outcome of the vote. No ~~member~~Member shall be entitled to vote in person or by electronic means at a meeting of ~~members~~Members if such ~~member~~Member has cast ~~her or his~~their vote in advance of the meeting by electronic ballot.

4.14 **Proposals at Annual Meetings**

Subject to compliance with section 163 of the Act, a ~~member~~Member entitled to vote at an annual meeting may submit to the ~~Corporation~~Association notice of any matter that the ~~member~~Member

proposes to raise at the annual meeting (a "proposal**Proposal**"). Any such proposalProposal may include nominations for the election of ~~directors~~Directors if the proposalProposal is signed by not less than five per cent (5%) of the ~~members~~Members entitled to vote at the meeting. Subject to the Act, the CorporationAssociation shall include the proposalProposal in the notice of meeting and if so requested by the ~~member~~Member, shall also include a statement by the ~~member~~Member in support of the proposalProposal and the name and address of the ~~member~~Member. The ~~member~~Member who submitted the proposalProposal shall pay the cost of including the proposalProposal and any statement in the notice of meeting at which the proposalProposal is to be presented unless otherwise provided by~~ ordinary resolution~~Ordinary Resolution of the ~~members~~Members present at the meeting.

~~4.15 **Resolution in Lieu of Meeting**~~

~~In accordance with and subject to the Act, a resolution in writing signed by all the members entitled to vote on that resolution at a meeting of members is as valid as if it had been passed at a meeting of the members, unless a written statement is submitted to the Corporation by a director or by the public accountant in relation to their resignation, removal or replacement. A copy of every resolution of the members shall be kept with the minutes of meetings of members.~~

SECTION 5 - DIRECTORS

5.01 **Number of Directors**

The ~~board~~Board shall consist of a number of ~~directors~~Directors between the minimum and maximum number of ~~directors~~Directors specified in the ~~articles~~Articles. The precise number of ~~directors~~Directors on the ~~board~~Board shall be determined from time to time by the ~~members~~Members by~~ ordinary resolution~~Ordinary Resolution, of, if the~~ ordinary resolution~~Ordinary Resolution empowers the ~~directors~~Directors to determine the number of ~~directors~~Directors, by resolution of the ~~board~~Board.

5.02 **Qualifications**

Each ~~director~~Director shall be an individual who is not less than 18 years of age. No person who has been found~~ by a court in Canada or elsewhere to be mentally incompetent~~to be incapable (as the term "incapable" is defined in the Act) or who has the status of a bankrupt shall be a ~~director~~Director. Only ~~members~~Members shall be eligible to be elected and hold office as ~~directors~~Directors of the CorporationAssociation, except for the position representing Students referred to in section 5.03(e),~~ and any retiring director who remains a member of the Corporation may be eligible for re-election~~. No person shall be eligible to hold more than one position on the ~~board~~Board.

5.03 **Composition of Board**

Directors shall, as far as practicable, be representative of the geographical regions of Canada, and of gender, sub-discipline interest and language groups. The ~~board~~Board shall be comprised as far as possible of the following ~~directors~~Directors, all of whom shall be elected by the ~~members~~Members except for those referred to in clause (a):

a) The ~~directors~~Directors appointed by the ~~board~~Board under section ~~5.07~~5.06 (if any);

b) Three (3) ~~directors~~Directors, of whom (i) one identifies as a scientist, (ii) one identifies as a practitioner, and (iii) one identifies as an educator;

c) Five (5) directorsDirectors-at-large (including the President and Immediate Past-President);

d) One (1) directorDirector who is nominated by and from among the Council of Sections; and

e) One (1) directorDirector who is nominated by and from among the Section on Students.

For greater certainty, if a Director described under section 5.03(b) or section 5.03(d) is appointed as the President or Immediate Past-President, they will then be deemed to be aDirector-at-large under section 5.03(c).

5.04 **Election of Directors and Term**

(a) Subject to the articlesArticles and byBy-laws, directorsDirectors shall be elected by the membersMembers by ordinary resolutionOrdinary Resolution at an annual meeting of membersMembers at which an election of directorsDirectors is required.

(b) The terms of office of directorsDirectors shall be established by ordinary resolutionOrdinary Resolution of the membersMembers provided that no directorDirector shall hold office for longer than a four (4) year term.

(c) If directorsDirectors are not elected at a meeting of membersMembers, the incumbent directorsDirectors shall continue in office until their successors are elected.

(d) Directors shall be eligible for re-election, except that no elected directorDirector shall serve for more than two (2) consecutive terms, unless authorized by the boardBoard in exceptional circumstances.

(e) The boardBoard shall establish a nominating committeeNominating Committee, the details of which shall be set forth herein and in the Operating Regulations. The nominating committeeGovernance Policies. The Nominating Committee will present a report to the membersMembers for the election of directorsDirectors and such report will be prepared in accordance with the requirements of this byBy-law and the Operating Regulations.Governance Policies.

5.05 **Absentee Voting by Mailed In Ballot or Electronic Ballot**

A Member entitled to vote at a meeting of Members may vote by mailed in ballot or may vote by means of a telephonic, electronic or other communication facility if the Corporation has a system set out in Operating Policies that enables the votes to be gathered in a manner that permits their subsequent verification, and permits the tallied votes to be presented to the Corporation without it being possible for the Corporation to identify how each Member voted.

5.05 5.06 **Nomination of Directors**

(a) General - Subject only to the Act and, the Articles, and the By-laws, only persons who are nominated in accordance with the following procedures shall be eligible for election as Directors of the Corporation. Nominations of persons for election to the Board may be made atby any Nominating Member in advance of any annual meeting of Members, or at any special meeting of Members if one of the purposes for which the special meeting was called is the election of Directors:

(i) by or at the direction of the Board, including pursuant to a notice of meeting;

(ii) by or at the direction or request of one or more Members pursuant to a proposal made in accordance with the Act, or a requisition of the Members made in accordance with the Act; or

( ) by any person as set forth herein. For purposes of this section 5.05, a nominating Member ("**Nominating Member**") means a Member: (A)

(i) who, at the close of business on the date of the giving of the notice provided for below and on the record date for notice of such meeting, is entered in the CorporationAssociation's membership records being entitled to vote at such meeting; and (B)

(ii) who complies with the notice procedures set forth below.

(b) Timely Notice - In addition to any other applicable requirements, for a nomination to be made by a Nominating Membervalid, the Nominating Member must have given timely noticesubmitted their nomination in proper written form to the Secretary of the Corporation at the registered office of the Corporation. To be timely, a Nominating Member's notice to the Secretary of the Corporation must be made:

(i) Association, in the case of an annual meeting of Membersaccordance with the instructions provided by the Association, not less than 30 nor more than 65 days prior to the date of the annual meeting of Members; provided, however, that in the event that the annual meeting of Members is to be held on a date that is less than 50 days after the date (the Notice Date) on which notice of the annual meeting was made, notice by the Nominating Member may be made not later than the close of business on the tenth (10th) day following the Notice Date; and

(ii) in the case of a special meeting (which is not also an annual meeting) of Members called for the purpose of electing Directors (whether or not called for other purposes) not later than the close of business on the fifteenth (15th) day

,

following the day on which the first public announcement of the date of the special meeting of Members was made;

in no event shall any adjournment or postponement of a meeting of Members or the announcement thereof commence a new time period for the giving of a Nominating Member's notice as described above.

(c) Proper Form - To be in proper written form, a Nominating Member's notice to the SecretaryAssociation must set forth:

(i) as to each person whom the Nominating Member proposes to nominate for election as a Director: (A) the name and address of the person; (B) the principal occupation or employment of the person; and (C) any other information confirming that the person meets all of the qualification requirements of Directors set out in the By-laws of the CorporationAssociation and such other applicable policies of the CorporationGovernance Policies; and

(ii) as to the Nominating Member giving the notice, (A) the name and the residential address of the person; (B) the principal occupation or employment of the person; (C) the class of membership of the person of the Corporation; and (DC) confirmation that the person has the right to vote at the meeting of Members where the election is to be held;

provided that the CorporationAssociation may also require any proposed nominee to furnish such other information, including a written consent to act, as may reasonably be required by the ~~Corporation~~Association to determine the eligibility of such proposed nominee to serve as a Director of the ~~Corporation~~Association.

~~(d)~~ ~~Eligibility~~Validity - ~~No person shall be eligible for election as a Director of the Corporation unless nominated in accordance with the provisions of this Section 5.06; provided, however, that nothing in this Section 5.06 shall be deemed to preclude discussion by a Member (as distinct from the nomination of Directors) at a meeting of Members of any matter in respect of which it would have been entitled to submit a proposal pursuant to the Act. The chair of the meeting~~The Nominating Committee shall have the power and duty to determine whether a nomination was made in accordance with the procedures ~~set out in~~requirements of this Section ~~5.06~~5.05 and, if any proposed nomination is not in compliance, to declare that such ~~defective~~ nomination shall be disregarded.

~~(e)~~ ~~Deliver~~Delivery of Notice - Notwithstanding any other provision of this By-law, notice given to the ~~Secretary of the Corporation~~Association pursuant to this Section ~~5.06~~5.05 may only be given by personal delivery, facsimile transmission or by email (at such email address as stipulated from time to time by the ~~Secretary of the~~ CorporationAssociation for purposes of this notice), and shall be deemed to have been given and made only at the time it is served by personal delivery, email (at the aforesaid address) or sent by facsimile transmission (provided that receipt of confirmation of such transmission has been received) to the ~~Secretary at the address of the~~ registered office of the CorporationAssociation; provided that if such delivery or electronic communication is made on a day which is a not a business day or later than 5:00 p.m. (EST) on a day which is a business day, then such delivery or electronic communication shall be deemed to have been made on the subsequent day that is a business day.

Notwithstanding the foregoing, the Board may, in its sole discretion, waive any requirement in this Section.

5.06 ~~5.07~~ Appointment of Directors and Term

Subject to the byBy-laws, following the conclusion of the annual general meeting of ~~members~~Members each year, the ~~board of directors~~Board may appoint one or more additional ~~directors~~Directors; however the total number of ~~directors~~Directors so appointed shall not exceed one-third (1/3) of the number of ~~directors~~Directors elected by the ~~members~~Members at the previous annual meeting of ~~members~~Members. The appointed ~~directors~~Directors will hold office as ~~directors~~Directors and officers for a term expiring not later than the close of the next annual meeting of ~~members~~Members. Appointed ~~directors~~Directors shall be eligible for re-appointment, except that no ~~director~~Director shall serve for more than six (6) consecutive terms, unless authorized by the ~~board~~Board in exceptional circumstances.

5.07 ~~5.08~~ Ceasing to Hold Office

A ~~director~~Director ceases to hold office when the ~~director~~Director dies, resigns, is removed from office by the ~~members~~Members in accordance with section ~~5.10~~5.09, or no longer fulfils all of the qualifications to be a ~~director~~Director set out in section 5.02 as determined in the sole discretion of the ~~board~~Board.

5.08 ~~5.09~~ Resignation

In accordance with and subject to the Act, a resignation of a directorDirector becomes effective at the time a written resignation is sent to the CorporationAssociation or at the time specified in the resignation, whichever is later.

5.09 5.10 Removal

In accordance with and subject to the Act, the membersMembers may, by ordinary resolutionOrdinary Resolution, passed at a meeting of membersMembers, remove any directorDirector from office before the expiration of the directorDirector's term and may elect a qualified individual to fill the resulting vacancy for the remainder of the term of the director so removed, failing which such vacancy may be filled by the board. The procedure to be followed may be set out in the Operating Regulations.

5.10 5.11 Filling Vacancies

In accordance with and subject to the Act and the articlesArticles, a quorum of the boardBoard may fill a vacancy in the boardBoard, except a vacancy resulting from an increase in the number or the minimum or maximum number of directorsDirectors, or from a failure of the membersMembers to elect the number of directorsDirectors required to be elected at any meeting of membersMembers. If there is not a quorum of the boardBoard, or if the vacancy has arisen from a failure of the membersMembers to elect the number of directorsDirectors required to be elected at any meeting of membersMembers, the boardBoard shall forthwith call a special meeting of membersMembers to fill the vacancy. If the boardBoard fails to call such meeting or if there are no directorsDirectors then in office, any memberMember may call the meeting. A directorDirector appointed or elected to fill a vacancy holds office for the unexpired term of their predecessor.

SECTION 6 - MEETINGS OF DIRECTORS

6.01 Calling of Meetings

Meetings of the boardBoard may be called by the President or any three (3) directorsDirectors at any time.

6.02 Notice of Meeting

Notice of the time and place for the holding of a meeting of the boardBoard shall be given in the manner provided in section 9.01 of this byBy-law to every director of the CorporationDirector not less than 48 hours before the time when the meeting is to be held. Notice of a meeting shall not be necessary if all of the directorsDirectors are present, and none objects to the holding of the meeting, or if those absent have waived notice of or have otherwise signified their consent to the holding of such meeting. Notice of an adjourned meeting is not required if the time and place of the adjourned meeting is announced at the original meeting. Unless the by law otherwise provides, noNo notice of meeting need specify the purpose or the business to be transacted at the meeting except that a notice of meeting of directors shall specify any matter referred to inif subsection 138(2136(3) of the Act that isrequires the purpose thereof or the business to be transacted to be dealt with atspecified in the meetingnotice.

6.03 Regular Meetings

The boardBoard may appoint a day or days in any month or months for regular meetings of the boardBoard at a place and hour to be named. A copy of any resolution of the boardBoard fixing

the place and time of such regular meetings of the ~~board~~Board shall be sent to each ~~director~~Director forthwith after being passed, but no other notice shall be required for any such regular meeting except if subsection 136(3) of the Act requires the purpose thereof or the business to be transacted to be specified in the notice.

6.04 **Participation at Meeting by Telephone or Electronic Means**

If all of the ~~directors~~Directors consent, a ~~director~~Director may, in accordance with the Regulations, participate in a ~~board~~Board meeting, by means of a telephonic, electronic or other communications facility that permits all participants to communicate adequately with each other during the meeting. A ~~director~~Director participating in the meeting by such means shall be deemed for the purposes of the Act to have been present at that meeting. A consent pursuant to this section may be given before or after the meeting to which it relates and may be given with respect to all meetings of the ~~board~~Board and committees of the ~~board~~Board.

6.05 **Votes to Govern**

At all meetings of the ~~board~~Board, every question shall be decided by a majority of the votes cast on the question. In case of an equality of votes, the chair of the meeting ~~in addition to an original vote~~ shall not have a second or casting vote.

6.06 **Quorum**

A quorum shall be a majority of the number of ~~directors~~Directors determined in accordance with section 5.01. For the purpose of determining quorum, a ~~director~~Director may be present in person, or, if authorized under this ~~by~~By-law, by teleconference and/or by other electronic means.

6.07 **Resolutions in Writing**

A resolution in writing, signed by all the ~~directors~~Directors entitled to vote on that resolution at a ~~board~~Board meeting, shall be as valid as if it had been passed at a ~~board~~Board meeting. A copy of every such resolution in writing shall be kept with the minutes of the proceedings of the ~~board~~Board or committee of ~~directors~~Directors.

6.08 **Board Committees and Advisory Bodies**

Subject to the Act, the ~~board~~Board may from time to time appoint any committee or other advisory body, as it deems necessary or appropriate for such purposes, subject to the Act, with such powers as the ~~board~~Board shall see fit. Any committee member may be removed by the ~~board~~Board. Unless otherwise determined by the ~~board~~Board, a committee shall have the power to fix its quorum at not less than a majority of its members, to elect its chairperson and to otherwise regulate its procedure. Terms of reference of all ~~committees, including standing~~Board committees, ~~nominations committee, committee on fellows and committee on elections~~ shall be ~~contained~~set out in the ~~Corporation's Operating Regulations~~Governance Policies.

6.09 **Partner Representatives**

(a) The ~~board~~ Board will designate up to four (4) organizations as partner organizations who each shall be entitled to appoint an individual to be its partner representative to represent it at meetings of the ~~board~~ Board.

(b) The partner representatives ~~shall~~may be ~~entitled~~invited to participate in ~~board~~Board meetings or portions thereof. For greater certainty, the partner representatives are not ~~directors,~~Directors and shall not be entitled to vote and shall not be counted for quorum purposes at any meeting of the ~~board~~Board.

(c) An individual shall become a partner representative effective upon the date of the appointment, and shall cease to be a partner representative upon the date that the appointment of ~~his or her~~ their successor is effective.

6.10 Disclosure of Interest

~~Every director~~Without limiting the requirements of the Act or any Governance Policies, every Director and officer shall disclose to the ~~Corporation~~Association the nature and extent of any interest that the ~~director~~Director or officer has in a material contract or material transaction, whether made or proposed, with the ~~Corporation~~Association, in accordance with the manner and timing provided in the Act.

SECTION 7 - OFFICERS

7.01 Appointment

The ~~board~~Board may designate the offices of the ~~Corporation~~Association, appoint officers, specify their duties and, subject to the Act, delegate to such officers the power to manage the affairs of the ~~Corporation~~Association. A ~~director~~Director may be appointed to any office of the ~~Corporation~~Association, except that the ~~director~~Director described in section 5.03(a) or 5.03(e) is not eligible to be appointed the President or Immediate Past-President. An officer may, but need not be, a ~~director~~Director unless these ~~by~~By-laws otherwise provide. The term of office of officers shall be one (1) year unless otherwise determined by the ~~board~~Board by resolution.

7.02 Description of Offices

Unless otherwise specified by the ~~board~~Board (which may, subject to the Act, modify, restrict or supplement such duties and powers), the offices of the ~~Corporation~~Association, if designated and if officers are appointed thereto, shall have the following duties and powers associated therewith, as well as such other duties and powers as the ~~board~~Board may specify from time to time:

(a) **Honorary President** – An Honorary President may be appointed ~~from time to time~~ by the ~~board~~Board for a period of one year. The duties of the Honorary President shall be determined by the ~~board~~Board.

(b) **President** - The President shall be a ~~member~~Member and ~~director~~Director of the ~~Corporation In the role of Chair of the board of directors the President is responsible~~

~~. , for the development and maintenance of policy affecting the affairs of the Corporation~~Association. The President shall act as ~~Chair~~chair of meetings of ~~members~~Members and the ~~board of directors thereof~~Board and shall see that all orders and resolutions of the Board are carried into effect. The President shall also be a member ex-officio of all committees of the ~~Corporation~~Board. The President shall have such other powers and shall perform such other duties as may from time to time be assigned to the President by resolution of the Board or as are set forth in the Governance Policies.

(c) **Immediate Past-President** – The Immediate Past-President shall be a ~~member~~Member and ~~director~~Director of the ~~Corporation~~Association and shall be appointed at the end of

the term of the incumbent President. The duties of the Immediate Past-President shall be determined by the ~~board~~Board.

(d) **Chief Executive Officer** – The Chief Executive Officer shall be appointed by the ~~board of directors~~Board to hold office until the termination of ~~his/her~~their contract and upon completion of all duties connected therewith.

The Chief Executive Officer shall have full authority subject to the authority of the ~~board of directors and the President~~Board, to manage and direct the affairs of the ~~Corporation~~Association. The Chief Executive Officer shall conform to all lawful orders given to ~~him/her~~them by the ~~board~~Board. The Chief Executive Officer shall give to the ~~directors~~Directors, to the officers or any one of them, from time to time, all information they may require regarding the affairs of the ~~Corporation~~Association. The Chief Executive Officer shall be responsible for the recording of all votes and minutes of all proceedings in the books to be kept for that purpose.

The Chief Executive Officer shall be responsible for keeping a register of ~~members~~Members and shall have custody of the corporate seal of the ~~Corporation~~Association. The Chief Executive Officer shall be responsible for preparing an annual budget for all accounts, for submission to the ~~board of directors~~Board for its consideration.

The Chief Executive Officer shall have custody of the corporate funds and securities and shall be responsible for keeping full and accurate accounts of receipts and disbursements in books belonging to the ~~Corporation~~Association, and shall be responsible for the deposit of all monies and other valuable effects in the name and to the credit of the ~~Corporation~~Association and in such depository as may be designated by the ~~board~~Board from time to time. The Chief Executive Officer shall be responsible for the disbursement of the funds of the ~~Corporation~~Association as may be ordered by the ~~board~~Board, and for taking proper vouchers for such disbursements, and shall render to the ~~board of directors~~Board whenever they may require it, an account of all transactions and of the financial position of the ~~Corporation~~Association.

The powers and duties of all other officers of the ~~Corporation~~Association shall be such as the terms of their engagement call for or the ~~board~~Board or President requires of them. The ~~board~~Board may from time to time and subject to the Act, vary, add to or limit the powers and duties of any officer.

7.03 **Vacancy in Office**

In the absence of a written agreement to the contrary, the ~~board~~Board may remove, whether for cause or without cause, any officer of the ~~Corporation~~Association. Unless so removed, an officer shall hold office until the earlier of:

(a) the officer's successor being appointed,

(b) the officer's resignation,

(c) such officer ceasing to be a ~~director~~Director (if a necessary qualification of appointment) or

(d) such officer's death.

If the office of any officer of the ~~Corporation~~Association shall be or become vacant, the ~~board~~Board may, by resolution, appoint a person to fill such vacancy.

SECTION 8 - SECTIONS

8.01 **Purpose**

Sections are the primary agents through which the particular and special needs of ~~members~~Members are met and interests are served. Those matters pertaining to the discipline of psychology as a whole are the responsibility of the ~~board of directors of the Corporation~~Board. The ~~board of directors~~Board may establish ~~Operating Regulations~~Governance Policies including rules and regulations concerning the formation, operation, conduct, and dissolution of Sections and may delegate the development and implementation of section operating regulations to the Chief Executive Officer.

8.02 **Formation**

Approval to establish a Section within the ~~Corporation~~Association may be granted by the ~~board~~Board when a group of at least 25 ~~Fellows and members~~Members of the ~~Corporation~~Association submit a petition to the ~~board~~Board which includes a statement of purpose and goals of the proposed Section.

8.03 **Dissolution and Restructuring**

A Section of the ~~Corporation~~Association may be dissolved by the ~~board~~Board (a) if the Section breaches any Governance Policies; (b) when the Section votes to request dissolution or when the number of ~~Fellows and members~~Members of the Section falls below 25 ~~Fellows and members~~Members. Without limiting the generality of the foregoing or any Governance Policies, the Board may, instead of dissolving a Section, remove and replace the members of the Section executive in the event that the Section breaches any Governance Policies. In the event that the number of ~~Fellows and members~~Members of the Section falls below 25 ~~Fellows and~~ Members, the Section may submit a petition to the ~~board of directors~~Board to stay dissolution for one year to enable the Section to re-establish the necessary number of ~~Fellows and~~ Members. If the Section is unable to achieve the required level within one year the Section shall be dissolved.

8.04 **Organization**

Within twelve months of obtaining approval to establish a Section (or within twelve months of the Board approving any amendments to the Model Terms of Reference described herein), the Section shall pass terms of reference in conformity with such Model Terms of Reference as may from time to time be approved by the ~~board of directors~~Board of the ~~Corporation~~Association and the Section terms of reference will take effect upon approval by the ~~board. The Section terms of reference will regulate among other things, the following:~~

~~(a) the admission of persons to the Section and the qualification for and the conditions of membership;~~

~~(b) the Section fees and dues of members;~~

~~(c) the suspension and termination of membership from the Section and by the member;~~

~~(d) the time for and the manner of election of directors of the Section; the election or appointment, functions, duties and removal of officers;~~

~~(e) the time and place and the notice to be given for the holding of meetings of members, the quorum at meetings; and~~

~~(f) the conduct in all particulars of the Section~~Board or its designate.

8.05 **Regulation of Section Affairs**

A Section may engage in activities of relevance to its members, and may adopt rules, regulations and policies for the management of its affairs ~~which~~that are not inconsistent with the ~~articles~~Articles, ~~by~~By-laws ~~or Operating Regulations of the Corporation. However~~, Governance Policies or section operating regulations of the Association. For greater certainty, nothing herein shall be construed as giving to a Section, the power and authority to:

(a) contract a loan, open a line of credit or issue a guarantee without the prior approval of the ~~board of directors~~Board;

(b) make any expense or disbursement, or incur any contractual liability which is in excess of the amounts approved by the ~~board of directors~~Board from time to time or as otherwise set out in the Governance Policies or section operating regulations; or

(c) make any public statements, issue position papers or policy statements, or otherwise represent or act on behalf of the ~~Corporation in any manner or thing, except with~~Section or the Association without the express written consent of the ~~board of directors of the Corporation~~Association.

8.06 **Dues and Fees Invoicing**

Sections may establish annual section dues which will be collected by the central office. Sections shall have the authority to solicit donations from their ~~members~~Members for specific identified purposes.

8.07 **Reports**

At least ~~four~~eight weeks before the ~~Annual General Meeting~~annual meeting of the ~~Corporation~~Association, the secretary of each Section shall submit an ~~Annual Report, which includes a financial statement,~~annual update to the ~~board~~Board of ~~directors of~~ the ~~Corporation. The financial statement shall include a budget for the ensuing year which shall be subject to approval~~Association that contains the information prescribed by the ~~board of directors~~Association.

8.08 **Council of Sections**

The chair of each Section shall form the Council of Sections of the ~~Corporation~~Association. The Council of Sections will provide guidance to the ~~board~~Board or the Chief Executive Officer from time to time and perform such other duties as may be specified in the ~~Operating Regulations~~Governance Policies.

~~8.09 **Powers**~~

~~A Section shall have the power to initiate and undertake activities of relevance to its members, to draft position papers on topics of relevance to the Section, to initiate policy statements in areas of expertise and to organize meetings. A Section may make specific representation to~~

~~external organizations or agencies only if it has received the approval of the board of directors to do so or it may recommend that the Corporation make such representation.~~

8.09 ~~8.10~~ **Access**

A Section shall have access to the ~~board of directors~~Board through the ~~director~~Director described at section 5.03(d) for consideration of issues of concern to the Section involving matters relevant to the relationship of the Section to the ~~Corporation~~Association as a whole or component thereof, or concerning agencies or activities external to the ~~Corporation~~Association.

SECTION 9 - NOTICES

9.01 **Method of Giving Notices**

Subject to section 4.01 and 6.02, any notice to be given (which term includes sent, delivered or served) pursuant to the Act, the ~~articles~~Articles, the ~~by~~By-laws or otherwise to a ~~member, director~~Member, Director, officer, member of a committee of the ~~board~~Board, or the public accountant shall be sufficiently given:

(a) if delivered personally to the person to whom it is to be given or if delivered to such person's address as shown in the records of the ~~Corporation~~Association or in the case of notice to a ~~director~~Director to the latest address as shown in the last notice that was filed by the ~~Corporation~~Association in accordance with the Act and received by Corporations Canada; or

(b) if mailed to such person at such person's recorded address by prepaid ordinary or air mail; or

(c) if sent to such person by telephonic, electronic or other communication facility at such person's recorded address for that purpose; or

(d) if provided in the form of an electronic document in accordance with the Act.

A notice so delivered shall be deemed to have been given when it is delivered personally or to the recorded address as aforesaid; a notice so mailed shall be deemed to have been given when deposited in a post office or public letter box; and a notice so sent by any means of transmitted or recorded communication shall be deemed to have been given when dispatched or delivered to the appropriate communication company or agency or its representative for dispatch. 21

The ~~Secretary~~Association may change or cause to be changed the recorded address of any ~~member, director~~Member, Director, officer, public accountant, or member of a committee of the ~~board~~Board in accordance with any information believed by the ~~Secretary~~Association to be reliable. The ~~declaration by the Secretary that notice has been given pursuant to this By law shall be sufficient and conclusive evidence of the giving of such notice. The~~ signature of any ~~director~~Director or officer of the ~~Corporation~~Association to any notice or other document to be given by the ~~Corporation~~Association may be written, stamped, type-written or printed or partly written, stamped, type-written or printed.

9.02 **Computation of Time**

If a given number of days' notice or notice extending over a period is required to be given under the ~~by~~By-laws, the day of service, posting or other delivery of the notice shall not, unless it is otherwise provided, be counted in such number of days or other period.

9.03 **Undelivered Notices**

If any notice given to a ~~member~~Member is returned on two consecutive occasions because such ~~member~~Member cannot be found, the ~~Corporation~~Association shall not be required to give any further notices to such ~~member~~Member until such ~~member~~Member informs the ~~Corporation~~Association in writing of ~~his or her~~their new address.

9.04 **Omissions and Errors**

The accidental omission to give any notice to any ~~member,~~Member, ~~director~~Director, officer, member of a committee of the ~~board~~Board or public accountant, or the non-receipt of any notice by any such person or any error in any notice not affecting its substance shall not invalidate any action taken at any meeting to which the notice pertained or otherwise founded on such notice.

9.05 **Waiver of Notice**

Any ~~member~~Member~~, director~~Director, officer, member of a committee of the ~~board~~Board or public accountant may waive or abridge the time for any notice required to be given to such person, and such waiver or abridgement, whether given before or after the meeting or other event of which notice is required to be given shall cure any default in the giving or in the time of such notice, as the case may be. Any such waiver or abridgement shall be in writing except a waiver of notice of a meeting of ~~members~~Members or of the ~~board~~Board or of a committee of the ~~board~~Board, which may be given in any manner.

SECTION 10 - BY-LAWS

10.01 **Amendment of Articles**

The Articles of the ~~Corporation~~Association may only be amended if the amendment is sanctioned by a ~~special resolution~~Special Resolution of the ~~members~~Members. Any amendment to the Articles is effective on the date shown in the certificate of amendment.

~~10.02 **By laws to be Confirmed by Special Resolution**~~

~~In accordance with the articles and subject to the Act, any by law, amendment or repeal of a by law shall require confirmation by special resolution of the members.~~

10.02 ~~10.03~~ **Effective Date of Board Initiated By-law, Amendment or Repeal**

Subject to the ~~articles~~Act and section ~~10.04~~10.03 of this ~~by~~By-law, the ~~board of directors~~Board may, by resolution, make, amend or repeal any ~~by~~By-laws that regulate the activities or affairs of the ~~Corporation~~Association. Any such ~~by~~By-law, amendment or repeal shall be effective from the date of the resolution of ~~directors~~Directors until the next meeting of ~~members~~Members where it may be confirmed, rejected or amended by the ~~members~~Members by ~~special resolution~~Ordinary Resolution. If the ~~by~~By-law, amendment or repeal is confirmed or confirmed as amended by the ~~members~~Members it remains effective in the form in which it was confirmed. The ~~by~~By-law, amendment or repeal ceases to have effect if it is not submitted to the ~~members~~Members at the next meeting of ~~members~~Members or if it is rejected by the ~~members~~Members at the meeting.

10.03 ~~10.04~~ **Effective Date of By-law, Amendment or Repeal under Subsection 197(1)**

A ~~by~~By-law made, amended or repealed under subsection 197(1) of the Act is effective from the date of the ~~special resolution~~Special Resolution of ~~members~~Members approving such ~~by~~By-law, amendment or repeal. ~~and need not be submitted to the board for approval. For greater certainty, under subsection 197(1) of the Act, a special resolution of the members is required to make any amendment to the articles or the by laws of the Corporation to:~~

~~(a) change the Corporation's name;~~

~~(b) change the province in which the Corporation's registered office;~~

~~(c) add, change or remove any restriction on the activities that the Corporation may carry on;~~

~~(d) create a new class of members;~~

~~(e) change a condition required for being a member;~~

~~(f) change the designation of any class of members or add, change or remove any rights and conditions of any such class;~~

~~(g) divide any class of members into two or more classes and fix the rights and conditions of each class;~~

~~(h) add, change or remove a provision respecting the transfer of membership;~~

~~(i) subject to section 133, increase or decrease the number of or the minimum and maximum number of directors fixed by the articles;~~

~~(j) change the statement of the purpose of the Corporation;~~

~~(k) change the statement concerning the distribution of property remaining on liquidation after the discharge of any liabilities of the Corporation;~~

~~(l) change the manner of giving notice to members entitled to vote at a meeting of members;~~

~~(m) change the method of voting by members not in attendance at a meeting of members; or~~

~~(n) add, change or remove any other provision that is permitted by the Act to be set out in the articles.~~

10.04 **Repeal of Previous By-laws**

All previous by-laws of the Association related to the subject matter of this By-law are repealed as of the effective date of this By-law. Such repeal shall not affect the validity of any act done or right or privilege, obligation or liability acquired or incurred under such By-law prior to its repeal. All Directors, officers, committee members, and persons acting under any by-law so repealed shall continue to act as if elected or appointed under the provisions of this By-law, and all resolutions of the Directors or Members with continuing effect passed under any repealed By-law shall continue to be good and valid except to the extent inconsistent with this By-law and until amended or repealed.

Adopted by the Special Meeting of the Canadian Psychological Association, ~~October~~ ~~19~~[•], ~~2018~~2025
